# Novel Application of UHPLC–MS, HRMS, and 2D‐NMR for Structural Elucidation of Eletriptan Hydrobromide and Its Novel Degradation Products

**DOI:** 10.1002/bmc.70347

**Published:** 2026-01-08

**Authors:** Dastagiri Reddy Bhuma, Venkata Kanaka Srivani Maddala, Suresh Salakolasu, Naresh Kumar Katari

**Affiliations:** ^1^ Department of Chemistry Vignan's Foundation for Science, Technology & Research Guntur Andhra Pradesh India; ^2^ Analytical Discovery Chemistry Aragen Life Sciences Pvt. Ltd. Hyderabad India; ^3^ School of Chemistry & Physics, College of Agriculture, Engineering & Science, Westville Campus University of KwaZulu‐Natal Durban South Africa; ^4^ A&B Laboratories (An AmSpec Group of Company) Houston Texas USA

**Keywords:** 2D‐NMR spectroscopy, degradation products, eletriptan, forced degradation study, high resolution mass spectrometry, UHPLC–MS spectrometry

## Abstract

A stability‐indicating UHPLC–MS method was developed to investigate the stress degradation behavior of eletriptan hydrobromide under ICH‐recommended acidic, alkaline, neutral, oxidative, thermal, and photolytic conditions. Significant degradation was observed only under acidic and oxidative stress. Four degradation products were isolated and structurally characterized using HRMS and NMR, including one novel dimeric species. The optimized UHPLC–MS method provided an 8‐min runtime with good resolution, sensitivity, and suitability for routine analysis. These results offer the first complete structural insight into the degradation pathways of eletriptan hydrobromide. The study supports improved quality control and long‐term stability assessment. The findings also bridge the knowledge gap left by earlier studies that reported unassigned degradation products. Three new degradation products were identified and isolated from oxidative degradation and a new degradation product isolated from the acid degradation, providing the first documented structural characterization of all four degradation products. Furthermore, we develop a UPLC MS method having 8‐min runtime and good peak shape and resolution by using Acquity BEH C18 column (100 × 2.1 mm, 1.7 μm) column.

**Abbreviations**
API: active pharmaceutical ingredient
DP: degradation products
ELE: eletriptan hydrobromide
ESI: electrospray ionization
FTIR: Fourier‐transform infrared spectroscopy
gHMBC: gradient hetero nuclear multiple bond coherence spectroscopy
HRMS: high‐resolution mass‐spectrometry
ICH: International Council for Harmonization
PDA: photo diode array detector
SQD: single quadrupole detector

## 1 Introduction

In order to clarify the drug's inherent stability, forced degradation studies are typically a component of the drug development strategy being implemented. As a result, these investigations are carried out in harsher and more dramatic circumstances than those typically employed for long‐term stability testing. The data acquired could aid in the development of appropriate analytical techniques and the identification of the drug breakdown route (Alsante et al. 2007; Jocić et al. 2009; El‐Bagary et al. 2012). A relatively new serotonin 5‐HT1B/1D receptor agonist called eletriptan hydrobromide (ELE) is used to treat acute migraine headaches. It is thought that ELE lessens vascular edema around the brain. This edema is linked to migraine attack‐related headache pain. Eletriptan inhibits the production of chemicals from nerve endings that increase pain and other symptoms, such as light and sound sensitivity and nausea. Eletriptan is believed to alleviate symptoms in part because of these actions. Its pharmacological actions include the constriction of cerebral blood vessels and the blocking of neuropeptide production, which ultimately results in pain relief (Hoskin et al. 2004; Durham et al. 2004).

In terms of chemistry, ELE is 3‐{[(R)‐1‐methyl‐2‐pyrrolidinyl] methyl}‐5‐[2‐(phenylsulfonyl) ethyl] indole hydrobromide. According to the literature, pharmacokinetic studies have used HPLC–MS/MS to quantitatively analyze eletriptan in human plasma (Ponnuru et al. 2011; Shah et al. 2002). A few studies of ELE tablet formulations have also examined the assay of ELE in the presence of associated organic contaminants (Shah et al. 2002; Sagirli et al. 2008; Zecevic et al. 2006). In both of the previously disclosed approaches, the chemical and physical stability of ELE is not reported. In the medication development cycle, stability studies depend on forced degradation (FD) research. Therefore, this study's primary goal was to conduct a thorough stress analysis on ELE by exposing it to a range of experimental settings. Acid, alkaline, neutral hydrolysis, oxidative, thermal, and photolytic degradation are all investigated. The study's primary goal was to speculate on the drug's potential breakdown mechanism. Numerous factors, including oxidation, photolysis, and hydrolysis (pH‐related degradation), affect the quality of molecules over time and with the strength of degradation events. When in solution or suspension, the test should additionally assess the drug substance's hydrolysis susceptibility over a broad pH range. According to the standards, photo stability testing needs to be a crucial component of stress testing (ICH Q1A (R2), Q2 (R1), Q3B (R2) 2006, and WHO 2007).

**FIGURE 1:**
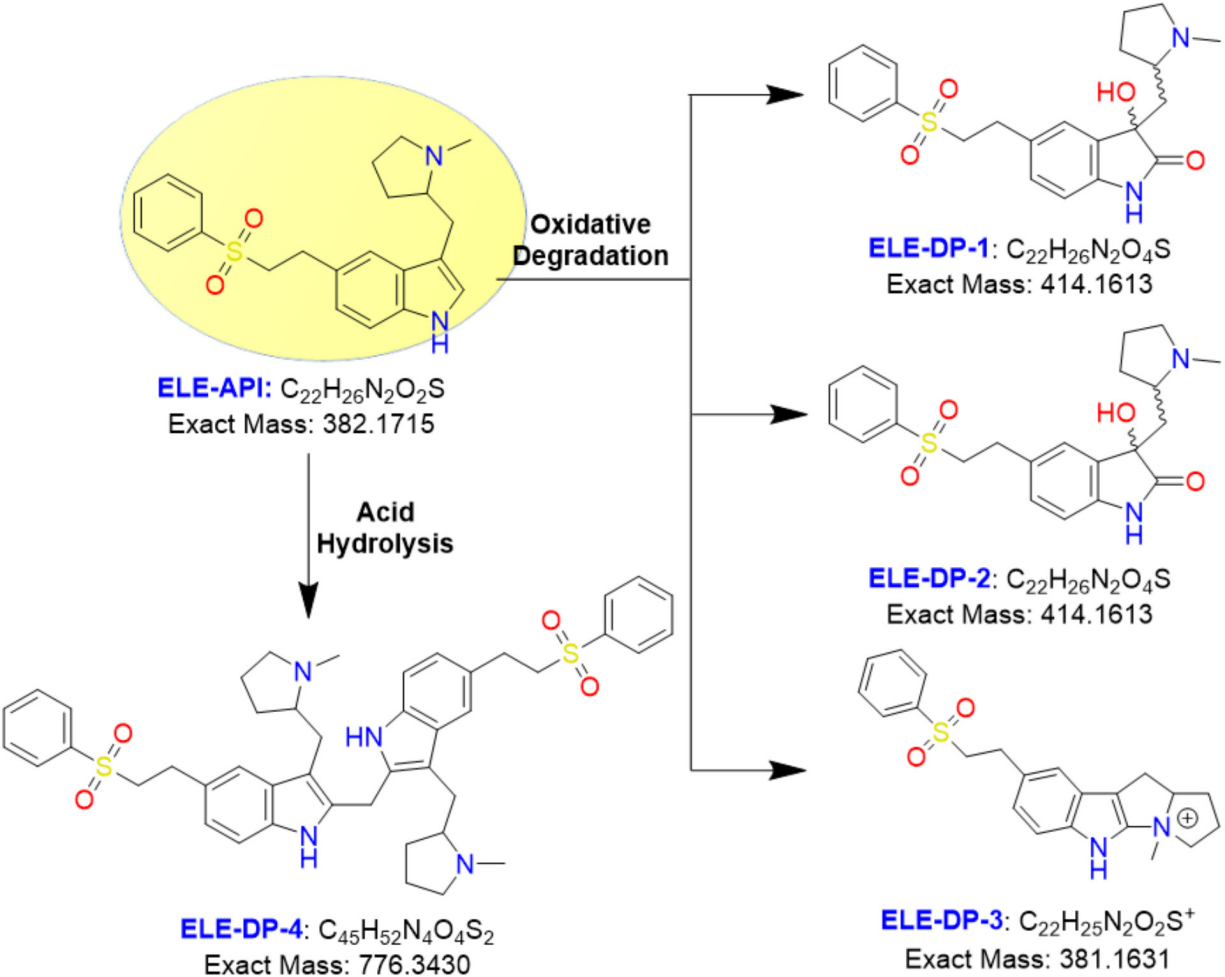
Structures of eletriptan and its degradation.

To identify and distinguish chromatographically degraded compounds, LC–MS/MS is one of the best technologies available. Similarly, for the purpose of clarifying structure, liquid chromatography–mass spectrometry (LCMS) might be helpful. Concerning the LC method for estimating ELE alone or in combination with other drugs in either API form or dosage form (Alsante et al. 2003; Xu et al. 2001; Rani et al. 2013), only a small number of the literature findings suggest a validated stability indicating assay method for ELE, while other literature suggests an HPLC method of analysis (Ngwa 2010; Salakolusu et al. 2023). However, there has not been any research up to this point that explains the breakdown products of eletriptan and their characterization. The nuclear magnetic resonance spectroscopy (NMR), IR, and high‐resolution mass spectrometry analyses of the degradation products were not included in any of the literature. In order to provide tangible proof of the degradation product structures, the current study developed a chromatographic method for ELE and its four degradation products, the UHPLC–MS method, which was well resolved in an 8‐min runtime. Additionally, preparative HPLC, high‐resolution mass‐spectrometry (HRMS), IR, and NMR (1D, 2D) experiments were conducted (Salakolusu et al. 2024; Kanagaddi et al. 2024). Figure 1 displayed all of the structures of eletriptan and its degrading impurity.

## 2 Materials and Methods

### 2.1 Chemicals and Reagents

The eletriptan hydrobromide API was a gift sample from one of the pharmaceutical organizations in Hyderabad. All of the specifics of the chemicals and reagents used in the study were obtained from Merck India Ltd. in Mumbai, India. These included acetonitrile (HPLC‐grade), trifluoro acetic acid, formic acid (LCMS‐grade), hydrogen peroxide (analytical grades), hydrochloric acid, sodium hydroxide, and DMSO‐d6 (NMR‐grade) from Cortec Net, water from a milli‐Q apparatus manufactured by Millipore in Amsterdam, the Netherlands, was utilized for the analysis. Water from a milli‐Q apparatus manufactured by Millipore in Amsterdam, the Netherlands, was utilized for the analysis.

### 2.2 Instrumentation and Software

For the current experiment, the LCMS equipment from Waters single quadrupole detector with Acquity UHPLC frontend and Maslynx 4.2 software are the analytical tools and supporting software used. Chromscope‐2.1 software, auto sampler 2707 with detector‐2489, and purification apparatus were from Waters binary module 2545. Topspin 4.09 software and an NMR instrument from Bruker Advance Neo 400 MHz. ir‐afinity‐1S infrared spectroscopy device from Shimadzu, along with Sartorius‐SQP‐F analytical balance and lab solution software.

#### 2.2.1 Ultrahigh‐Performance Liquid Chromatography–Mass Spectrometry

Acquity UHPLC and the diode array detector (DAD) front‐end were used in conjunction with a waters single quadrupole detector (SQD2) to accomplish liquid chromatography separation. The waters single quadrupole SQ Detector‐2 mass spectrometer, which uses an electrospray ionization (ESI) source and operates in both negative and positive polarity, was used to analyze the mass. MS optimization was carried out using the 100–2000 Da (Da) scan mode. A capillary voltage of 3.5 kV and a source temperature of 140°C were established. A temperature of 350°C was chosen for desolvation. It was decided to set the desolvation gas flow at 650 L/h and the cone gas flow at 50 L/h. MassLynx 4.1 Application Manager was used to operate the liquid chromatography–mass spectrometer device. The samples were run with an injection volume of 0.2 μL and a chromatographic duration of 8.1 min while being kept at 10°C.

##### 2.2.1.1 UHPLC–MS Method Development (Optimization of Chromatographic Conditions)

The chromatographic conditions involved the use of Waters Acquity UPLC BEH C18 (100 × 2.1 mm, 1.7 μm) column, CORTECS UPLC C18, 2.1 × 30 mm, 1.6 μm and Kinetex C18, 50 × 2.1 mm, 1.7 μm. The mobile phase comprised mobile phase A: 0.05% TFA in water and mobile phase B: 0.05% TFA in acetonitrile.

**FIGURE 2:**
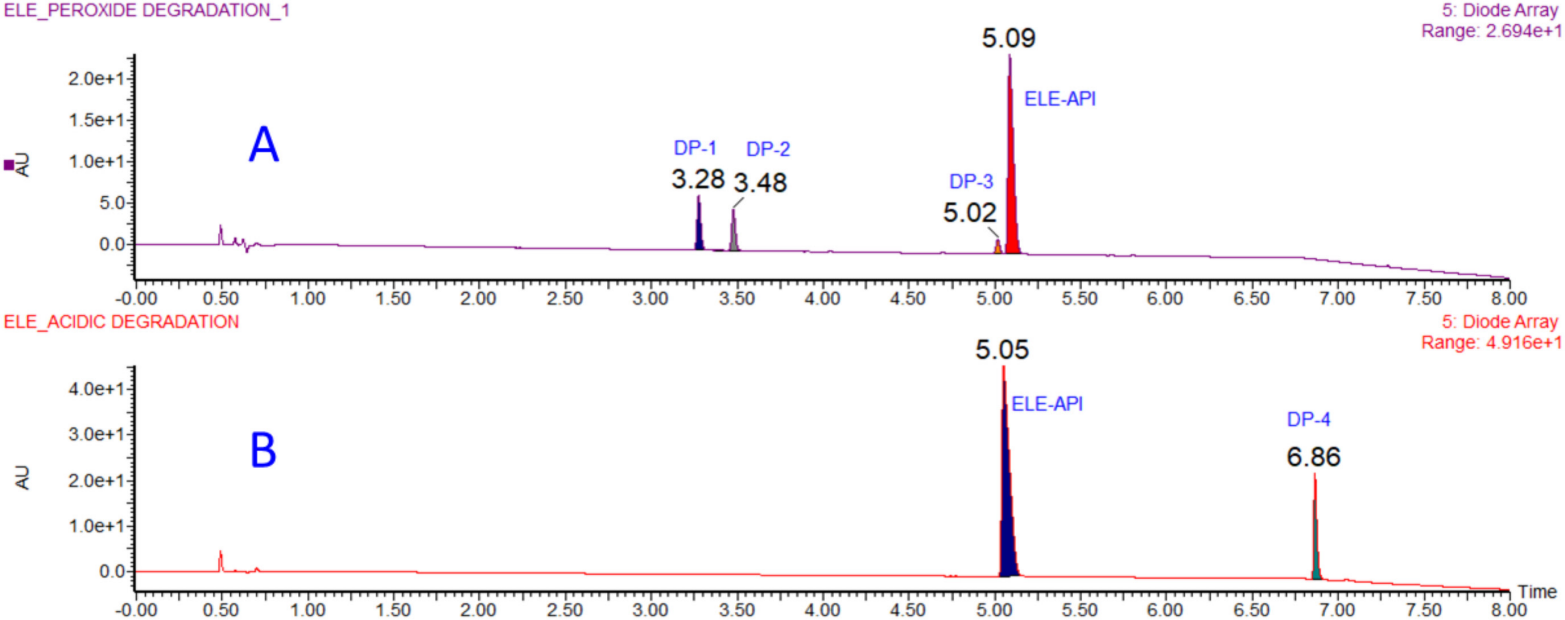
Degradation behavior of eletriptan peroxide (A) and acid (B) hydrolysis.

To find a viable, reproducible, and reliable approach, multiple UHPLC columns with different buffers were tested to optimize resolution. In numerous trials, 0.05% TFA buffer with Acquity BEH C18 produced positive and encouraging results. The optimum separation of all degradation impurities and API was achieved in Acquity BEH C18, 100 × 2.1 mm, 1.7 μm, with 0.05% TFA in aqueous (mobile phase A) and 100% acetonitrile (mobile phase B), flow rate 0.4 mL/min; column temperature 50°C; with binary gradient time/B Conc (%): 0/10, 6/35, 8/100, and 8.1/10. All peaks were separated with good resolution and better peak shape in this condition. The final method development trace with all the peaks was shown in Figure 2.

#### 2.2.2 High‐Resolution Mass‐Spectrometry

**FIGURE 3:**
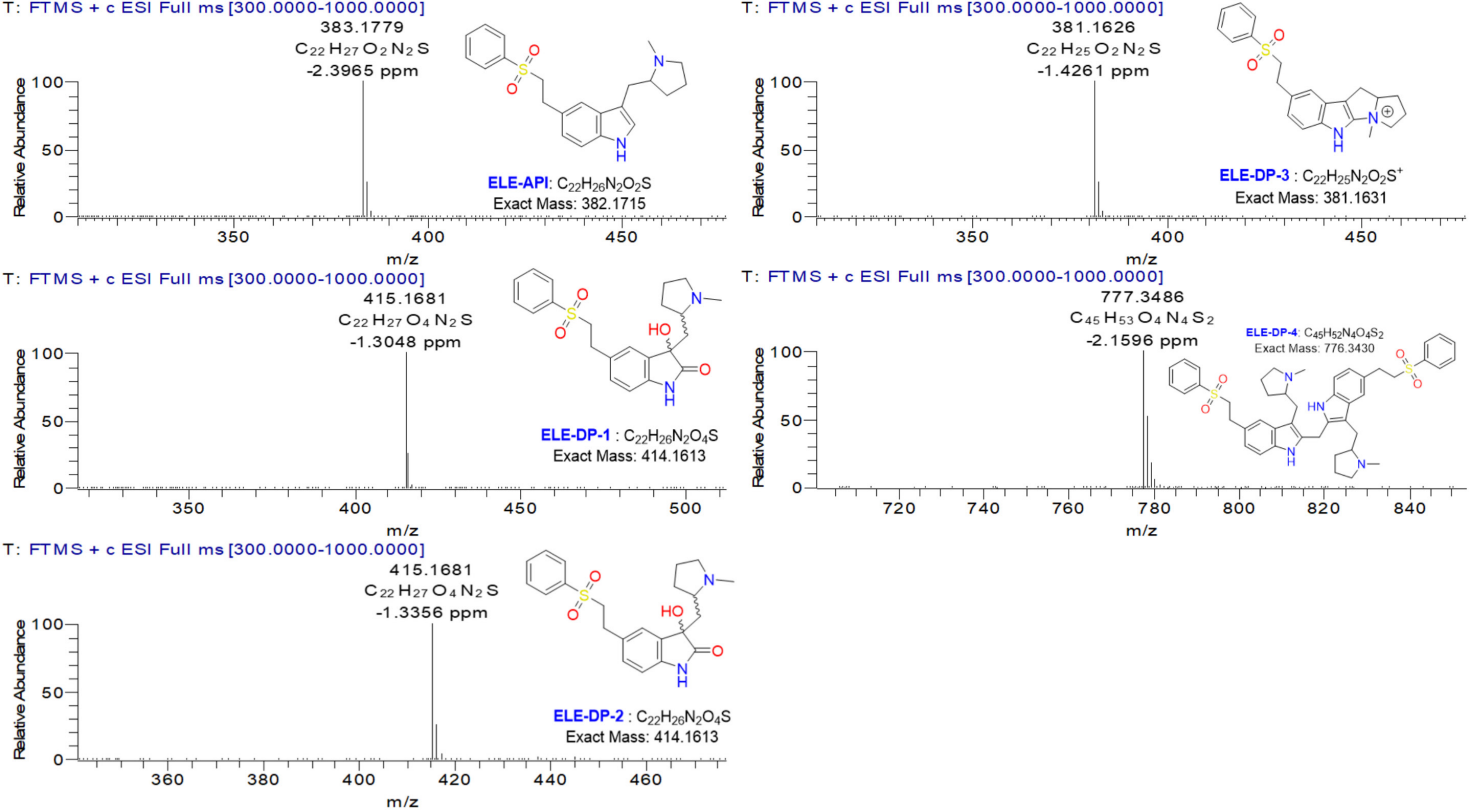
HRMS analysis data for eletriptan and its degradation products.

The samples were analyzed using a UHPLC Dionex Ultimate 3000 front‐end with a PDA detector and an ESI source equipped with a Thermo Q Exactive orbitrap MS. The instrument source specifications that were employed were as follows: spray voltage: 3.5 KV, insource CID: 40 eV, capillary temperature: 270°C, auxiliary gas flow rate: 14, and auxiliary gas heater temperature: 440°C. Sheath gas flow rates are 53 and sweep gas flow rates are 3. Mass data were gathered using Xcalibur software. Reserpine, which has a monoisotopic mass of 608.2734 Da, was used as a reference to confirm the correctness of the mass analyzer. Chromatographic conditions similar to LC–MS were employed. The HRMS data for every degradation product is shown in Figure 3. Table 1 shows the mass data for each degradation product acquired from the HRMS study.

**TABLE 1:** HRMS and FT‐IR data for eletriptan and its degradation products.

HRMS data				
Products name	Formula	m/z calculated	m/z obtained	Mass error(ppm)
Eletriptan	C_22_H_26_O_2_N_2_S	382.1715	383.1779	−2.3965
DP‐1	C_22_H_26_O_4_N_2_S	414.1613	415.1681	−1.3048
DP‐2	C_22_H_26_O_4_N_2_S	414.1613	415.1681	−1.3356
DP‐3	C_22_H_25_O_2_N_2_S+	381.1631	381.1626	−1.4261
DP‐4	C_45_H_53_O_4_N_4_S_2_	776.3430	777.3486	−2.1596

FT‐IR data				
Assignment	O‐H/N‐H stretching	C=O stretching	C‐O/C‐N stretching	C‐H bending
Region (cm^−1^)	3700–3300	1760–1630	1370–1000	1620–1450
Eletriptan	3372.59	1647.24	1144.7	1482.3
DP‐1	3472.93	1732.11	1055.08	1457.67
DP‐2	3742.93	1679.06	1201.67	1499.68
DP‐3	3504.72	1683.89	1201.67	1450.49
DP‐4	3381.27	1680.03	1302.94	1448.57

#### 2.2.3 Preparative HPLC

This semipreparative HPLC has a ChromScope‐2.1 software, a 2545 pump module, a 2707 sample manager with auto fraction collector‐III, and a 2489 dual UV detector. Using a mobile phase of 0.1% v/v TFA in water and 0.1% TFA in acetonitrile, the degradation products were isolated using a Gemini NX C18, 5 μm, 250 × 30 mm, with a flow rate of 18 mL/min. A Lyofreeze Lyophilizer was utilized to lyophilize every separated fraction.

#### 2.2.4 Nuclear Magnetic Resonance Spectroscopy

Eletriptan and degradation products' ^1^H, ^13^C, and 2D NMR spectra were captured in DMSO‐d6 solvent using a Bruker Advance Neo 400 MHz NMR equipment fitted with a 5 mm Broadband Observe probe (BBO) and a Z‐gradient shim system, with sensitivities of 480:1 and 225:1. The ^1^H and ^13^C septet signals of DMSO‐D6 were referenced to the TMS (tetramethyl silane) signal at zero and 39.5 ppm, respectively.

#### 2.2.5 FT‐IR Spectroscopy

To determine which functional groups, such as methyl and bromine, were present in the compounds, the Shimadzu IR‐Afinity‐1S model with Lab solutions software was utilized. The samples were made into pellets using KBr as a dispersion medium.

### 2.3 Degradation Samples Preparation for Purification

Initially, different stress parameters—such as acid, basic, neutral, oxidation, thermal, and photolytic conditions—were used in accordance with ICH stability criteria. Eletriptan's acid degradation tests were carried out in compliance with ICH guidelines. Using 600 mg of eletriptan in 1 N HCl and 600 mg of eletriptan in 30% hydrogen peroxide, an acid research was conducted for 48 h at 60°C under reflux conditions, with a total degradation of roughly 25%. For reverse phase purification, the reaction mass that resulted from the acid stress study was neutralized using a saturated base solution, lyophilized to create a solid sample, and then dissolved in 5.0 mL of mobile phase.

## 3 Results and Discussion

To ascertain the outcomes of each stress study, individual samples were examined using mass spectrometry. The experimental section describes the study circumstances for each constraint and all of the degradants that developed over time. UHPLC–MS, HRMS, FT‐IR, and NMR (1D and 2D) techniques were used to identify, isolate, and characterize four significant degradation products.

### 3.1 Degradation Trend of Eletriptan

Stress factors such as acidic, basic, neutral, oxidative, thermal, and photolytic conditions were used in accordance with ICH stability criteria (ICH 2003, 2005, 2006; WHO 2007). The chemical demonstrated great stability in neutral, alkaline, thermal, and photolytic environments and did not create any degradation products when exposed to a variety of FD stress settings. This indicates that eletriptan is stable under the aforementioned conditions. The medication was discovered to be susceptible to acid hydrolysis and oxidation. Acid hydrolysis and peroxide degradation chromatograms are given in Figure 2, and Table 2 presents comprehensive degradation conditions and outcomes. Three degradation products are produced during peroxide degradation (DP‐1, DP‐2, and DP‐3). DP‐4 is the only degradation product that formed in acidic conditions; Figure 1 displays the conformed structures of all the DPs.

**TABLE 2:** Eletriptan forced degradation studies.

Conditions	% Area of degradation product				
	DP‐1	DP‐2	DP‐3	DP‐4	API
Eletriptan API	—	—	—		99
Acid (1 Mol.L^−1^ HCl stirring at 60°C up to 48 h)	—	—	—	17.08	82.47
Base (1 Mol.L^−1^ NaOH stirring at 60°C up to 48 h)	—	—	—		99
Neutral (Water stirring at 60°C up to 48 h)	—	—	—		99
Oxidation (30% H_2_O_2_ stirring at rt up to 48 h)	13.6	10.16	3.82		71.61
Thermal (explore to 100°C up to 48 h)	—	—	—		99
Photolytic (explore at 254 nm for 48 h)	—	—	—		99

The ELE standard solution (10 mg/mL) was diluted with 30% hydrogen peroxide solution after 1 mL of the solution was placed into a 10‐mL volumetric flask. At room temperature, the mixture was swirled around. The solution was taken in milliliters and diluted with 1:1 v/v ACN and water to make 10 mL. To determine the degradation behavior, it was taken for more research in order to reach a final concentration of 100 μg/mL. In a UV chamber, a 25‐mg sample of eletriptan was exposed to 254 nm UV light for 48 h in order to cause photodegradation. For thermal degradation, a sample of 25 mg of eletriptan was stored at 100°C for 48 h. The deterioration behavior was then observed in subsequent investigations.

A standard stock solution of ELE (10 mg/mL) was made in ACN and water (1:1 v/v) for the neutral, acid, and base hydrolysis investigation. A 10‐mL volumetric flask was filled with 1 mL of the ELE stock solution. Next, apply makeup to the appropriate level using water for neutral degradation, 1 N HCl for acidic degradation, and 1 N NaOH for alkaline degradation (the identical process was initially carried out with 0.1 N NaOH and 0.1 N HCl). At 60°C, the solution was continuously stirred. After neutralizing 1 mL of the solution, it was moved to a 10‐mL volumetric flask. After that, water was added to the volume to achieve a final concentration of 100 μg/mL. In order to determine the degradation behavior, it was taken for additional research.

The stability of ELE in the aforementioned conditions is confirmed by the fact that the compound is extremely stable in neutral, basic hydrolysis, photolytic, and thermal environments and did not exhibit any degradation. It was discovered that the medication was susceptible to acid hydrolysis and peroxide. As a consequence, up to 48 h of reflux at 60°C under stirring circumstances showed about 15%–20% degradation in acid (1 N HCl). Table 2 and Figure 2 for the degradation chromatograms of peroxide and acid hydrolysis show specific degradation circumstances and outcomes. Three DPs were generated during peroxide hydrolysis, and one DP (DP‐4) was formed during acid hydrolysis; nonetheless, Figure 1 displays the verified structures of each DP.

### 3.2 Isolation of Degradation Products

A considerable percentage of impurity formation > 5% was observed under acidic hydrolysis conditions. Gemini C18, 5 μ, 250 × 30‐mm purification column with 0.1% TFA in aqueous and 0.1% TFA in acetonitrile was used as a mobile phase for purification. Following consecutive injections of crude sample solutions, the fractions were collected by UV and LC–MS mass confirmation. Separated fractions of different degradation products were then lyophilized to produce a free solid.

### 3.3 Structural Confirmation of Degradation Products

**FIGURE 4:**
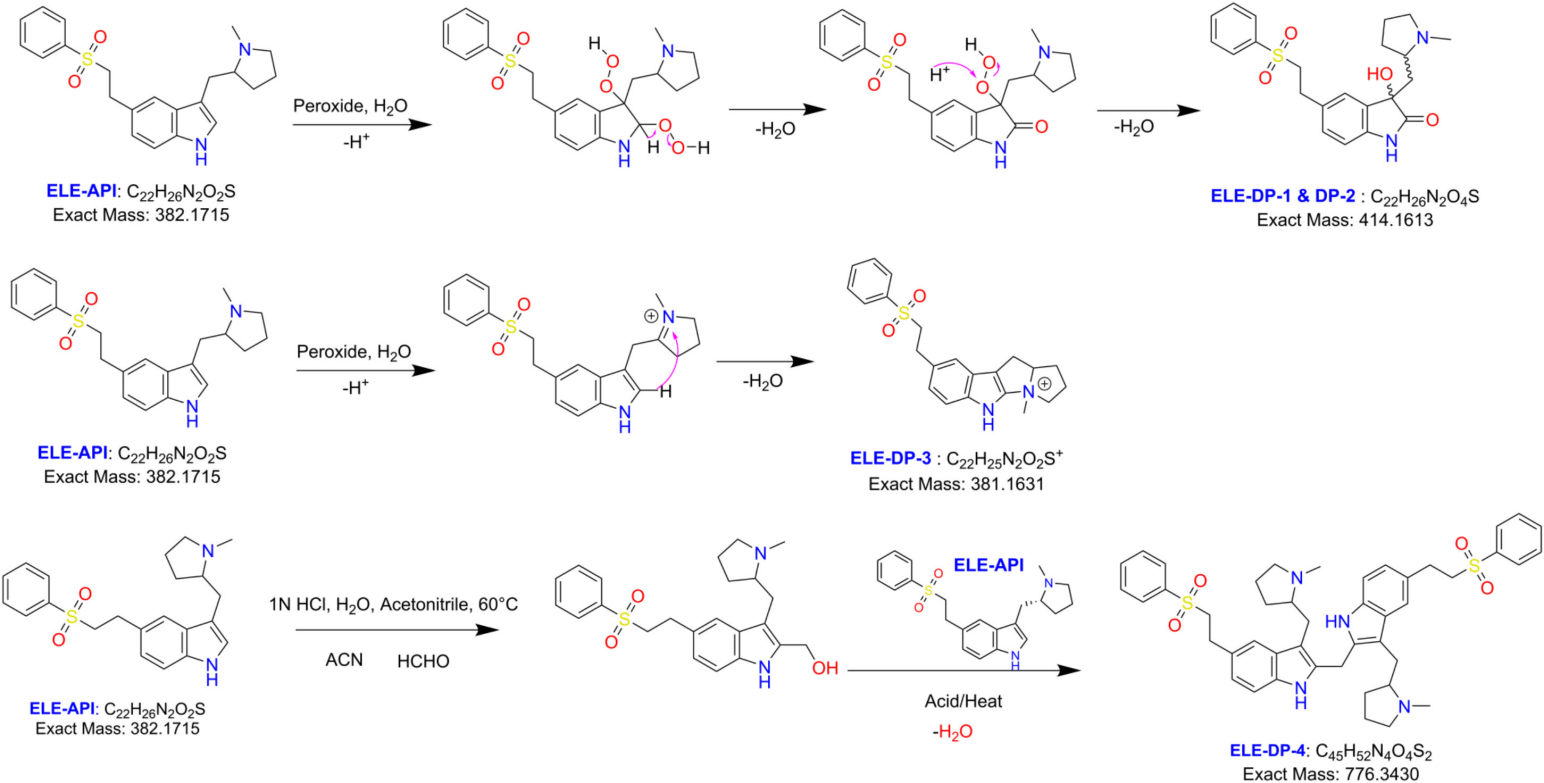
Proposed peroxide and acid degradation mechanism for eletriptan.

All analytical data were recorded for future use in order to acquire the structural information of the eletriptan API. Under positive ESI‐MS settings, HRMS determined the [M + H]^+^ ion to be 383.1779 Da, indicating that the chemical formula for eletriptan is C_22_H_27_O_2_N_2_S. The confirmed ELE results were indicated in the HRMS spectrum (Figure 2, Table 1), IR (Table 1), and 2D‐NMR (Table 3). The proposed molecular mechanism that explains how eletriptan breaks down in both peroxide and acidic hydrolytic conditions is shown in Figure 3. The structural conformation of ELE as determined by 2D‐NMR is displayed in Figure 1 and S1. HRMS and 2D‐NMR are utilized to achieve the structural conformation of all DP. This comparison of structural elucidation data is used to describe all other degradant products. The degradation products via the proposed degradation mechanism are shown in Figure 4. Eletriptan HBr shows two primary degradation pathways depending on the applied stress conditions. Under acidic hydrolysis, protonation of the ether linkage weakens the adjacent C–O bond, leading to cleavage and formation of a distinct acid‐derived degradant. Under oxidative conditions, the tertiary amine undergoes initial N‐oxidation, forming an N‐oxide intermediate. Further oxidation and cleavage of the benzenesulfonamide side chain generate two additional oxidative degradants. With prolonged exposure, reactive oxidative intermediates undergo intermolecular coupling, resulting in a unique dimeric degradation product, which has not been previously reported. All final structures are supported by HRMS and confirmed through full NMR structural characterization.

**TABLE 3:** Relative NMR assignments for eletriptan and its degradation products.

ELE‐API				ELE‐DP1				ELE‐DP‐2				ELE‐DP‐3				ELE‐DP‐4			
Atom no	Type of atom	1H chemical shift (ppm)	13C chemical shift (ppm)	Atom no	Type of atom	1H chemical shift (ppm)	13C chemical shift (ppm)	Atom no	Type of atom	1H chemical shift (ppm)	13C chemical shift (ppm)	Atom no	Type of atom	1H chemical shift (ppm)	13C chemical shift (ppm)	Atom no	Type of atom	1H chemical shift (ppm)	13C chemical shift (ppm)
		Coupling const (J)				Coupling const (J)				Coupling const (J)				Coupling const (J)				Coupling const (J)	
1,3	CH	7.67 (t,J = 7.60 Hz,2H)	129.43	1,3	CH	7.68 (t,J = 8.0 Hz,2H)	129.47	1,3	CH	7.68 (t,J = 7.60 Hz,2H)	129.46	1,3	CH	7.66 (t,J = 8.0 Hz,2H)	129.4	1,3,28,30	CH	7.68 (t,J = 8 Hz,4H)	129.47
2	CH	7.76 (tt,J = 7.60 Hz,1.2 Hz,1H)	133.8	2	CH	7.77 (tt,J = 7.20 Hz,2H,1H)	133.87	2	CH	7.77 (t,7.20 Hz,1H)	133.86	2	CH	7.75 (td,J = 7.20 Hz,2 Hz,1H)	133.77	2,29	CH	7.77 (t,d = 7.60 Hz,2H)	133.85
4,6	CH	7.96 (dd,J = 8.40 Hz,1.2 Hz,2H)	127.71	4,6	CH	7.93 (dd,J = 8.40 Hz,1.2 Hz,2H)	127.68	4,6	CH	7.93 (d.J = 7.60,2H)	127.7	4,6	CH	7.93 (dd,J = 8.4,1.6 Hz,2H)	127.72	4,6,31,33	CH	7.95 (dd,8.80,2.0 Hz,4H)	127.71
5	C	—	138.97	5	C	—	138.92	5	C	—	138.93	5	C	—	138.99	5,32	C	—	138.95
10	C	—	127.59	10	C	—	131.36	10	C	—	131.15	10	C	—	129.85	10,37	C	—	127.8
11	CH	6.92 (dd,J = 8.40 Hz,1.60 Hz)	121.94	11	CH	7.07 (dd,J = 8 Hz,1.60 Hz,1H)	129.47	11	CH	7.06 (d,J = 7.60 Hz,1H)	129.38	11	CH	7.09 (dd,J = 8.8,2 Hz,1H)	124.11	11,38	CH	6.86 (dd,J = 8.0 Hz,0.1.20 Hz,2H)	121.51
12	CH	7.24 (d.J = 8.0 Hz,1H)	111.59	12	CH	6.72 (d,8.0 Hz,1H)	109.82	12	CH	6.72 (d,7.60 Hz,1H)	109.78	12	CH	7.37 (d,J = 8.4 Hz,1H)	113.35	12,39	CH	7.16 (d,J = 8.40 Hz,2H)	111.2
13	C	—	135.11	13	C	—	139.9	13	C	—	139.83	13	C	—	138.51	13,40	C	—	134.38
14	C	—	126.86	14	C	—	131.41	14	C	—	131.68	14	C	—	121.52	14,41	C	—	127.86
15	CH	7.40 (s,1H)	117.65	15	CH	7.28 (d, J = 0.80 Hz,1H)	124.21	15	CH	7.24 (s,1H)	124.37	15	CH	7.35 (s,1H)	119.41	15,42	CH	7.34 (s,2H)	117.19
16	NH	10.93 (d,J = 1.60 Hz,1H)	—	16	NH	10.40 (s,1H)	—	16	NH	10.42 (s,1H)	—	16	NH	12.32 (s,1H)	—	16,43	NH	10.77 (s,2H)	—
17	C	—	108.62	17	C	—	73.48	17	C	—	73.71	17	C	—	108.88	17,44	C	—	105.74
18	CH	7.26 (s,1H)	124.35	18	CO	—	178.56	18	CO	—	178.35	18	C	—	140.53	18,45	C	—	133.69
19	CH_2_	2.89 (dd,J = 14 Hz,9.6 Hz,1H),	26.01	19	CH_2_	2.05 (dd,J = 14 Hz,9.20 Hz,1H),	38.12	19	CH_2_	1.90, 2.28 (m,2H)	38.38	19	CH2	2.88 (dd,J = 14.4 Hz,3.20 Hz,1H),	29.44	19,46	CH_2_	2.87 (dd,J = 13.6 Hz,3.20 Hz,2H),	24.57
		3.27 (dd,J = 14.8 Hz,5.2 Hz,1H)				2.26 (dd,J = 13.6 Hz,2.80 Hz,1H)								3.48 (dd,J = 14.40 Hz, 8.0 Hz)				3.21 (dd,J = 13.60Hsz,4.40 Hz,2H)	
20	CH	3.63 (m,1H)	68.22	20	CH	3.30 (broad signal, 1H)	64.13	20	CH	3.45 (broad peak,1H)	64.07	20	CH	5.23 (m,1H)	90.83	20,47	CH	3.31 (broad peak,1H)	68.21
21	CH_2_	1.72, 2.02 (m,2H)	29.52	21	CH_2_	1.63, 2.14 (m,2H)	30.68	21	CH_2_	1.55, 1.95 (m,2H)	31.04	21	CH_2_	2.02, 2.54 (m,2H)	32.21	21,48	CH_2_	1.63, 1.82 (m,4H)	29.19
22	CH_2_	1.91 (m,2H)	20.92	22	CH_2_	1.82, 1.90 (m,2H)	21.38	22	CH_2_	1.88 (m,2H)	21.42	22	CH_2_	1.92, 2.26 (m,2H)	25.28	22,49	CH_2_	1.82 (m,4H)	20.62
23	CH_2_	3.06.3.61 (m,2H)	55.78	23	CH_2_	2.97, 3.49 (m,2H)	54.75	23	CH_2_	3.01, 3.52 (m,2H)	54.81	23	CH_2_	3.85, 3.96 (m,2H)	66.4	23,50	CH_2_	2.96, 3.60 (m, 4H)	55.55
24	NH	9.53 (braod hump)	—	24	NH	9.45 (braod signal, 1H)	—	24	NH	9.72 (broad hump)	—	25	NCH_3_	3.59 (s,3H)	53.26	25,52	NCH_3_	3.82 (s,6H)	39.1
25	NCH_3_	2.84 (s,3H)	39.27	25	NCH3	2.78 (s,3H)	39.19	25	NCH_3_	2.79 (s,3H)	39.23	26	CH_2_	3.64 (m,2H)	55.98	26,53	CH_2_	3.61 (m,4H)	56.31
26	CH_2_	3.65 (m,2H)	56.26	26	CH_2_	3.59 (m,2H)	55.52	26	CH_2_	3.59 (m,2H)	55.55	27	CH_2_	2.95 (m,2H)	28.32	27,54	CH_2_	2.93 (m,4H)	28.51
27	CH_2_	2.95 (m,2H)	28.44	27	CH_2_	2.85 (m,2H)	27.79	27	CH_2_	2.84 (m,2H)	27.84					55	CH_2_	4.25 (s,4H)	23.27
				28	OH	6.25 (broad peak,1H)	—	28	OH	6.36 (broad hump, 1H)	—								

### 3.4 Degradation Product DP‐1 Characterization

The eletriptan treatment with peroxide resulted in degradation product 1 and 2. These degradation products were isolated and have the same molecular weight and molecular formula; the first isolated peak is named DP1, and the second one is DP‐2. By HRMS, compound mass was confirmed as 415.1681 units with molecular formula C_22_H_26_N_2_O_2_S (Figure 3). The mass of these degradation compounds is 32 units higher than that of the parent compound, suggesting a possible oxidation involving two moles. NMR studies were crucial in determining the oxidation position within the molecule.

The proton NMR spectrum shows 27 protons (including salt), with 11 protons from the aromatic region and 16 from the aliphatic region. The most downfield protons in the molecule are H16 and H24, appearing at 10.40 and 9.45 ppm, respectively. These are followed by the phenylsulfonyl and oxindole ring protons, which appear between 6.50 and 8.0 ppm. The key proton of the molecule, a tertiary alcohol, appears at 6.25 ppm. Aliphatic protons are present between 1.0 and 4.0 ppm. The molecule has 22 carbons in its skeleton, but due to the symmetry of the phenylsulfonyl ring, only 20 signals appear in the spectrum. Comparing the carbon results with the parent compound, two major changes were observed in the indole ring.

1. The C17 carbon in the parent compound is at 108.62 ppm, while in DP‐1 it is at 73.48 ppm.
2. The C18 carbon in the parent compound resonates at 124.35 ppm, while in DP‐1 it is at 178.56 ppm.

**FIGURE 5:**
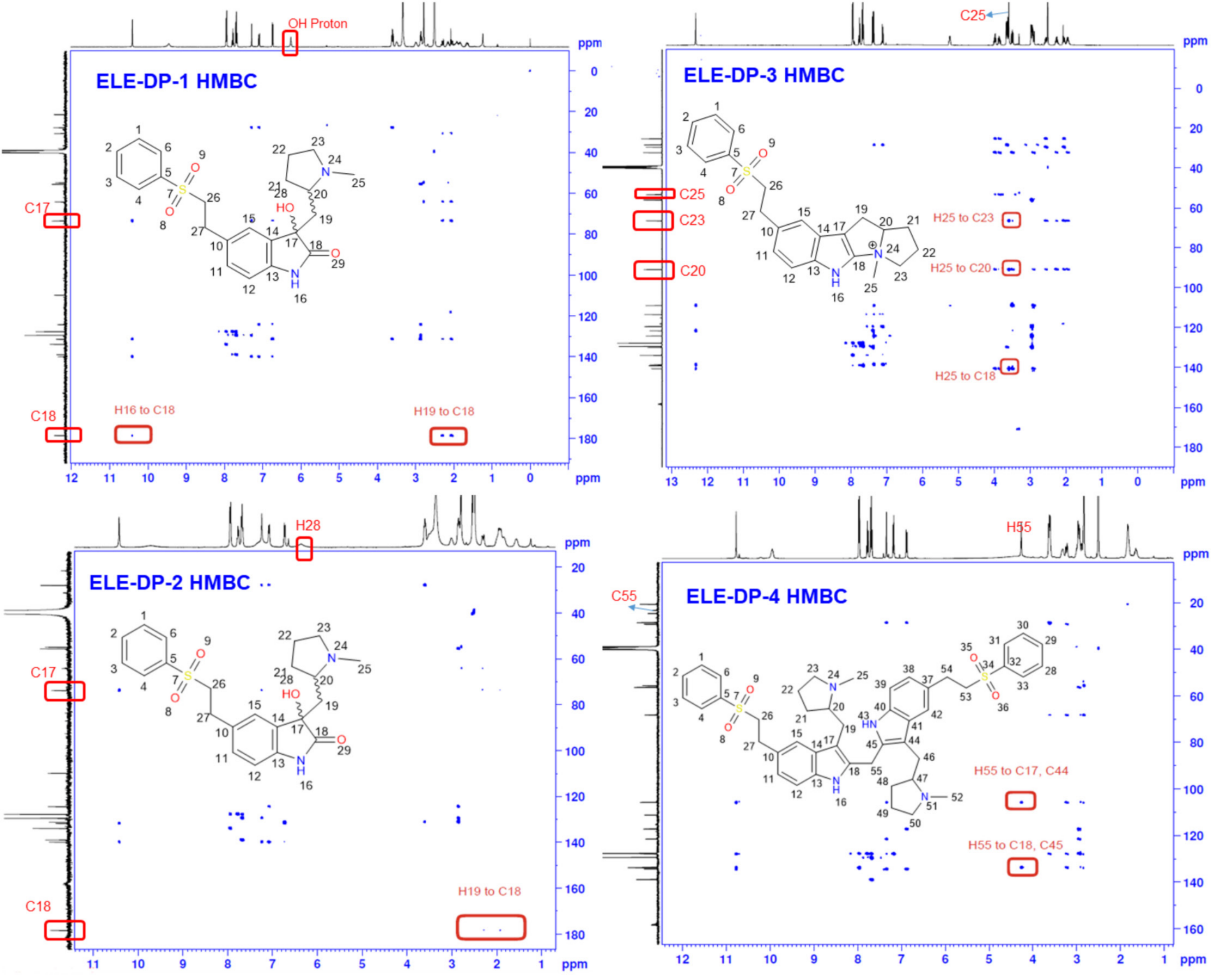
Key NMR confirmations for eletriptan degradation products.

HSQC experiment data revealed that one ‐CH proton is less than in the parent compound (Indole ‐CH) due to oxidation at the C18 carbon. HSQC also helped in peak assignments of the pyrrolidine ring. Finally, the HMBC experiment resolved the structure of the DP‐1 compound with a few key correlations: (A) H19 methylene and H16 protons show connectivity to the C18 carbon. (B) H15 and H16 protons show connectivity to the C17 carbon. These correlations confirmed that the indole ring carbons (C17, C18) are oxidized under peroxide conditions, forming 2‐oxindole. Consequently, a new chiral center was generated in the 2‐oxindole ring. The structure of DP‐1 was confirmed as 2‐((3‐hydroxy‐2‐oxo‐5‐(2‐(phenylsulfonyl) ethyl) indolin‐3‐yl) methyl)‐1‐methylpyrrolidin‐1‐ium and the 2D NMR spectrum was depicted in Figures 5 and S2. The chemical shifts were in Table 3 and the proposed degradation mechanism was shown in Figure 4.

### 3.5 Degradation Product DP‐2 Characterization

The molecular formula, molecular weight, and NMR results (2D correlations) of ELE‐DP1 and ELE‐DP2 were identical. Further structural confirmation revealed that DP‐1 and DP‐2 are diastereomers, which accounts for the slight differences observed in their chemical shifts. To determine the structure of ELE‐DP2, HRMS and NMR tests were performed. HRMS confirmed the compound's mass as 415.1681 units with the molecular formula C_22_H_26_N_2_O_2_S (Figure 3).

Similar to the DP‐1 compound, DP‐2 also has 27 protons. Notable observations from the ^1^H NMR data include the presence of a tertiary alcohol proton at 6.36 ppm and the absence of the indole ‐CH proton. The remaining pyrrolidine and phenylsulfonyl ring protons are present as usual in the parent compound. The ^13^C NMR data confirm the number of carbons in the molecule and their chemical environment, providing insights into the molecule's functionality. Like the DP‐1 compound, DP‐2 also shows two major changes in the carbon skeleton: The appearance of a newly formed amidic carbonyl group at 178.35 ppm (C18 carbon). The tertiary alcohol‐attached carbon (C17) appeared at 73.71 ppm. The rest of the aromatic carbons appear between 100 and 140 ppm, while the aliphatic carbons appear between 20 and 70 ppm. The total carbons in the spectrum were 22. The HSQC experiment reconfirms the absence of one indole ‐CH proton, as one correlation is missing in DP‐2 compared to the parent compound. All other aliphatic protons attached to carbons are distinguished using the HSQC data. Key correlations in the HMBC data include H19 methylene protons to C17 and C18 carbons, reinforcing the oxidation position on C17 and C18 carbons and formation of 2‐oxindole in the molecule. Finally, the structure of DP‐2 is confirmed as 2‐((3‐hydroxy‐2‐oxo‐5‐(2‐(phenylsulfonyl)ethyl)indolin‐3‐yl)methyl)‐1‐methylpyrrolidin‐1‐ium with a new chiral center in the molecule (C17 position), as shown in the Figures 5 and S3. The chemical shifts were in Table 3 and the proposed degradation mechanism was shown in Figure 4.

### 3.6 Degradation Product DP‐3 Characterization

With the peroxide degradation conditions, ELE results in the DP‐3 product. HRMS analysis determined the compound's mass and the molecular mass as 381.1626 Da [M + H]^+^ and molecular formula C_22_H_25_N_2_O_2_S. The mass of the ELE‐DP‐3 compound is 2 units less than that of the parent compound. To accurately determine the structure of DP‐3, 1D NMR and 2D NMR experiments were conducted.

The ^1^H NMR spectrum confirms the presence of 25 protons in the molecule. Similar to the DP‐1 and DP‐2 compounds, the indole methine proton is missing in DP‐3, and the pattern of aliphatic protons differs from the parent compound. The indole ‐NH proton appears at 12.32 ppm, aromatic protons (phenylsulfonyl and indole ring) appear between 7.0 and 8.0 ppm, and aliphatic protons appear between 1.50 and 5.50 ppm. Notably, the H25 methyl proton appears at 3.59 ppm, while the H20 methine and H23 methylene protons appear at 5.23, 3.85, and 3.96 ppm, respectively.

The ^13^C NMR data confirm the presence of 22 carbons in the molecule, with 20 signals appearing due to the symmetry in the phenylsulfonyl ring. The formation of a fused bicyclic ring with quaternary nitrogen in the molecule causes significant changes in the chemical shifts of aliphatic carbons, particularly C20, C23, and C25. The HSQC experiment was influential in assigning all the methine, methylene, and methyl carbons. The HSQC data confirmed the C20‐CH carbon at 90.83 ppm, C23‐CH_2_ at 66.40 ppm, and C25‐CH_3_ at 53.26 ppm. The remaining carbon assignments are provided in the Table 3.

Lastly, the HMBC experiment data strengthened the DP‐3 structure with the following correlations: (A) H25 methyl protons show connectivity to C18, C20, and C23 carbons. (B) H19 methylene protons show connectivity to C14, C17, C18, C20, and C21 carbons.

The key highlight of the HMBC data, which strongly supports the structure of DP‐3, is the connectivity of the H25 methyl protons to the C18 carbon. This is possible only with the formation of a fused bicyclic ring on the indole ring carbon. Lastly, the structure of DP‐3 is confirmed as 4‐methyl‐8‐(2‐(phenylsulfonyl)ethyl)‐2,3,4,5,10,10a‐hexahydro‐1H‐pyrrolizino[3,2‐b]indol‐4‐ium, as presented in the Figures 5 and S4. The chemical shifts were in Table 3 and the proposed degradation mechanism was shown in Figure 4.

### 3.7 Degradation Product DP‐4 Characterization

DP‐4 is the isolated product from the reaction mixture of ELE acid hydrolysis. Among the four degradation products, DP‐4 stands out due to its higher molecular weight, which is attributed to its dimerized nature. The HRMS analysis results helped predict the compound's molecular formula and molecular weight. Consequently, the molecular mass was confirmed as 777.3486, and the molecular formula as C_45_H_53_O_4_N_4_S_2_ (Figure 3).

Primarily, 1D NMR and 2D NMR results were crucial in determining the precise structure of DP‐4. Initially, 1D NMR studies (^1^H and ^13^C) were conducted, followed by HSQC and HMBC experiments. In proton NMR, the total proton count is 54 along with salt proton, with four labile protons (H16, H43 at 10.77 ppm, and H24, H51 at 9.94 ppm). 16 protons appeared in the downfield region (6.0–8.0 ppm), and 34 protons were observed in the upfield region (1.0–5.0 ppm). In the aliphatic region, a new methylene group proton was observed at 4.25 ppm, which was not present in the parent compound or any other degradation products. In ^13^C NMR, 21 carbon signals were noticed out of 45 carbons, reiterating the dimerization of the molecule. Among these, a new carbon signal appeared in the spectrum at 23.27 ppm. Aryl ring carbons were observed between 100 and 140 ppm, while aliphatic carbons resonated between 20 and 70 ppm. In the HSQC experiment, the newly appeared methylene carbon chemical shift was confirmed at 23.27 ppm (H55 → C55), which also assisted in the peak assignments of all other proton‐attached carbons in the molecule. In the HMBC experiment, a key correlation was observed with the H55 methylene (4.25 ppm) showing connectivity to C18, C45, and C17, C44 carbons. Additionally, indole ‐NH protons (H16, H43), H19, and H46 methylene protons also showed common connectivity to these carbons. This information extensively confirms that the methylene group was formed on the indole ring carbons positioned between C18 and C45 carbons, resulting in a dimer product, as shown in the Figure 5. The chemical name of DP‐4 is bis(3‐((1‐methylpyrrolidin2‐yl)methyl)‐5‐(2‐(phenylsulfonyl)ethyl)‐1H‐indol‐2‐yl)methane. The key NMR spectrum was shown in Figure 5 and the complete analysis data were shown in Figure S5. The chemical shifts were in Table 3 and the proposed degradation mechanism was shown in Figure 4.

## 4 Conclusion

Eletriptan's deterioration in stressful conditions was examined in accordance with ICH guidelines. The API was subjected to oxidative, photolytic, neutral, acidic, alkaline, and thermolytic degradation conditions. The drug material remained stable and no degradation products were seen under base, neutral hydrolysis, thermal, and photolytic conditions. Nevertheless, four novel degradation products were created under peroxide and acid hydrolysis conditions. These degradants are produced by oxidation, cyclization, and dimerization, all of which are vulnerable to acid hydrolysis. All degradation products were thoroughly separated and characterized using analytical methods such as LCMS and NMR (1D and 2D investigations). An additional layer of information was supplied by FT‐IR data to confirm the structures. The four DPs are all brand‐new products that have not been covered in any books. Each of the four DPs is a brand‐new product with no published documentation. The present study provides a thorough structural interpretation of eletriptan and its four degradation products using LCMS, HRMS, FT‐IR, and 2D‐NMR analyses. It also discusses the creation of a well‐resolved LC–MS method for separating these degradation products.

## Author Contributions

**Mr. Dastagiri Reddy Bhuma:** investigation, formal analysis, methodology, software, and writing – original draft. **Prof. Venkata Kanaka Srivani Maddala:** data curation, resources, and supervision. **Dr. Suresh Salakolasu:** visualization and validation. **Dr. Naresh Kumar Katari:** conceptualisation and writing – review and editing.

## Supporting information


**Figure S1:** Structural characterization data of eletripton.
**Figure S2:** Structural characterization data of ELE‐DP‐1.
**Figure S3:** Structural characterization data of ELE‐DP‐2.
**Figure S4:** Structural characterization data of ELE‐DP‐3.
**Figure S5:** Structural characterization data of ELE‐DP‐4.

## Data Availability

The data that support the findings of this study are available within the article. The raw data of this study are available from the corresponding author, upon reasonable request.

## References

[bmc70347-bib-0001] Alsante, K. M. , A. Ando , R. Brown , et al. 2007. “The Role of Degradant Profiling in Active Pharmaceutical Ingredients and Drug Products.” Advanced Drug Delivery Reviews 59, no. 1: 29–37. 10.1016/j.addr.2006.10.006. 17187892

[bmc70347-bib-0002] Alsante, K. M. , T. D. Hatajik , D. Santafianos , T. S. Sharp , and L. L. Lohr . 2003. “Impurity Isolation and Characterization Case Studies.” In Handbook of Isolation and Characterization of Impurities in Pharmaceuticals, 361–400. Academic Press (Elsevier).

[bmc70347-bib-0003] Durham, P. L. , P. X. Dong , K. T. Belasco , et al. 2004. “Neuronal Expression and Regulation of CGRP Promoter Activity Following Viral Gene Transfer Into Cultured Trigeminal Ganglia Neurons.” Brain Research 997, no. 1: 103–110. 10.1016/j.brainres.2003.11.005. 14715155

[bmc70347-bib-0004] El‐Bagary, R. I. , N. G. Mohammed , and H. A. Nasr . 2012. “Two Chromatographic Methods for the Determination of Some Antimigraine Drugs.” Analytical Chemistry Insights 7: ACI‐S8864. 10.4137/ACI.S8864. PMC3362328 22654488

[bmc70347-bib-0005] Hoskin, K. L. , G. A. Lambert , C. Donaldson , and A. S. Zagami . 2004. “The 5‐Hydroxytryptamine1b/1D/1F Receptor Agonists Eletriptan and Naratriptan Inhibit Trigeminovascular Input to the Nucleus Tractus Solitarius in the cat.” Brain Research 998, no. 1: 91–99. 10.1016/j.brainres.2003.11.018. 14725972

[bmc70347-bib-0006] ICH . 2003. “Stability Testing of New Drug Substances and Products Q1A(R2)”.

[bmc70347-bib-0007] ICH . 2005. “Validation of Analytical Procedures: Text and Methodology Q2(R1)”.

[bmc70347-bib-0008] ICH . 2006. “Impurities in New Drug Products Q3B (R2)”.

[bmc70347-bib-0009] Jocić, B. , M. Zečević , L. Živanović , A. Protić , M. Jadranin , and V. Vajs . 2009. “Study of Forced Degradation Behavior of Eletriptan Hydrobromide by LC and LC–MS and Development of Stability‐Indicating Method.” Journal of Pharmaceutical and Biomedical Analysis 50, no. 4: 622–629. 10.1016/j.jpba.2009.01.034. 19250786

[bmc70347-bib-0010] Kanagaddi, R. , V. Chintala , S. S. Nannapaneni , et al. 2024. “Isolation and Identification of Forced Degradation Products of Febuxostat.” Separation Science Plus 7, no. 5: 2300237. 10.1002/sscp.202300237.

[bmc70347-bib-0011] Ngwa, G. 2010. “Forced Degradation as an Integral Part of HPLC Stability‐Indicating Method Development.” Drug Delivery Technology 10, no. 5: 56–59.

[bmc70347-bib-0012] Ponnuru, V. S. , B. R. Challa , and R. Nadendla . 2011. “Quantitative Analysis of Eletriptan in Human Plasma by HPLC‐MS/MS and Its Application to Pharmacokinetic Study.” Analytical and Bioanalytical Chemistry 401, no. 8: 2539–2548. 10.1007/s00216-011-5341-4. 21892641

[bmc70347-bib-0013] Rani, N. U. , R. S. Rao , K. Sarswathi , and T. E. G. K. Murthy . 2013. “Development and Validation of Stability‐Indicating RP‐HPLC Method for Analysis of Eletriptan.” International Journal of Pharmaceutical, Chemical & Biological Sciences 3, no. 3: 708–715.

[bmc70347-bib-0014] Sagirli, O. , A. Önal , and D. Şensoy . 2008. “LC Assay of Eletriptan in Tablets and In Vitro Dissolution Studies.” Chromatographia 68: 269–273. 10.1365/s10337-008-0673-8.

[bmc70347-bib-0015] Salakolusu, S. , N. K. Katari , G. V. R. Sharma , et al. 2023. “Identification, Isolation, and Structural Characterization of Novel Forced Degradation Products of Ertugliflozin.” Scientific Reports 13, no. 1: 9472. 10.1038/s41598-023-36289-9. 37301855 PMC10257675

[bmc70347-bib-0016] Salakolusu, S. , N. K. Katari , G. V. R. Sharma , et al. 2024. “Utilizing Cutting‐Edge Analytical Techniques for the Identification, Isolation, and Structural Characterization of Donepezil HCl Forced Degradation Products.” Microchemical Journal 207: 111665. 10.1016/j.microc.2024.111665.

[bmc70347-bib-0017] Shah, A. K. , S. C. Harris , C. Greenhalgh , and J. Morganroth . 2002. “The Pharmacokinetics and Safety of Single Escalating Oral Doses of Eletriptan.” Journal of Clinical Pharmacology 42, no. 5: 520–527. 10.1177/00912700222011571. 12017346

[bmc70347-bib-0018] WHO . 2007. “Draft Stability Testing of Active Pharmaceutical Ingredients and Pharmaceutical Products”.

[bmc70347-bib-0019] Xu, X. , M. G. Bartlett , and J. T. Stewart . 2001. “Determination of Degradation Products of Sumatriptan Succinate Using LC‐MS and LC‐MS‐MS.” Journal of Pharmaceutical and Biomedical Analysis 26, no. 3: 367–377. 10.1016/s0731-7085(01)00409-5. 11489382

[bmc70347-bib-0020] Zecevic, M. , B. Jocic , S. Agatonovic‐Kustrin , and L. Zivanovic . 2006. “Validation of an HPLC Method for the Simultaneous Determination of Eletriptan and UK 120.413.” Journal of the Serbian Chemical Society 71, no. 11: 1195–1205. 10.2298/jsc0611195z.

