# Supplementary material for: Novel Application of UHPLC–MS, HRMS, and 2D‐NMR for Structural Elucidation of Eletriptan Hydrobromide and Its Novel Degradation Products
Source: Biomed Chromatogr. 2026 Jan 8;40(2):e70347. doi: 10.1002/bmc.70347 (PMC12783014; doi:10.1002/bmc.70347)
Supplement: Supplementary file 1 — Figure S1: Structural characterization data of eletripton. Figure S2: Structural characterization data of ELE‐DP‐1. Figure S3: Structural characterization data of ELE‐DP‐2. Figure S4: Structural characterization data of ELE‐DP‐3. Figure S5: Structural characterization data of ELE‐DP‐4. [file BMC-40-e70347-s001.docx]

**Novel Application of UHPLC-MS, HRMS, and 2D-NMR for Structural Elucidation of Eletriptan Hydrobromide and its Novel Degradation Products.**

Dastagiri Reddy Bhuma^a,b^, Venkata Kanaka Srivani Maddala^a^*, Suresh Salakolasu^b^, Naresh Kumar Katari^,c^*.

**SUPPORTING INFORMATION**

**Eletriptan Analytical data:**

ELE-API structure

Relative NMR assignments for Eletriptan:

| **Atom No** | **Atom Symbol** | **Chemical Shift (PPM) & Coupling Const(J-Hz)** | | **Atom No** | **Atom Symbol** | **Chemical Shift (PPM) & Coupling Const(J-Hz)** | |
| --- | --- | --- | --- | --- | --- | --- | --- |
|  |  | **^1^H** | **^13^C** |  |  | **^1^H** | **^13^C** |
| 1 | C | - | 149.09 | 15 | CH_2_ | 1.26,1.67(m,2H) | 37.4 |
| 2 | C | - | 155.25 | 16 | CH | 1.70(m,1H) | 31.6 |
| 3 | CH | 7.09(s.1H) | 108.14 | 17 | CH_2_ | 1.79,1.64(m,2H) | 29.04 |
| 4 | C | - | 148.65 | 18,20 | N-CH_2_ | 2.90,3.30(m,4H) | 51.25,51.31 |
| 5 | C | - | 128.27 | 21 | CH_2_ | 1.56,1.90(m,2H) | 28.1 |
| 6 | CH | 7.05(s.1H) | 103.91 | 22 | N-CH_2_ | 4.25(d,4.80Hz,2H) | 58.87 |
| 7 | CH_2_ | 2.63.3.22(m,2H) | 32.59 | 23 | C | - | 129.85 |
| 8 | CH | 2.65(m,1H) | 44.37 | 24,28 | CH | 7.64(m,2H) | 131.47 |
| 9 | CO | - | 206.23 | 25,27 | CH | 7.45(m,2H) | 128.66 |
| 11 | OCH_3_ | 3.78(s,3H) | 55.57 | 26 | CH | 7.45(m,1H) | 129.33 |
| 13 | OCH_3_ | 3.86(s,3H) | 55.94 |  |  |  |  |
|  |  |  |  |  |  |  |  |


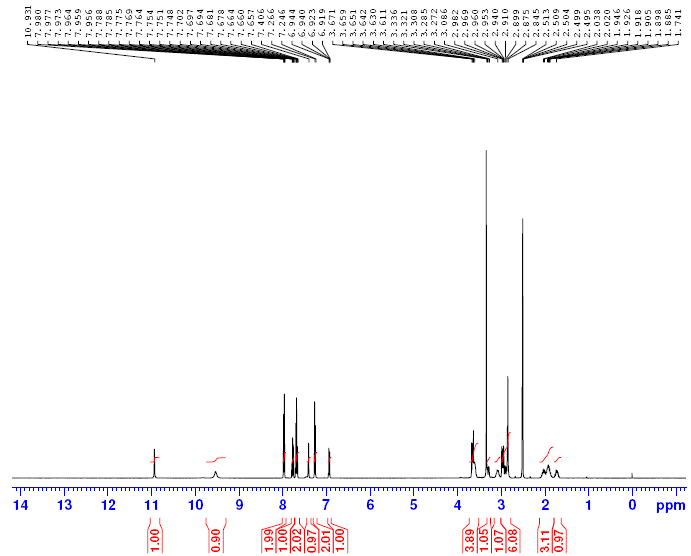


ELE-API ^1^H NMR data


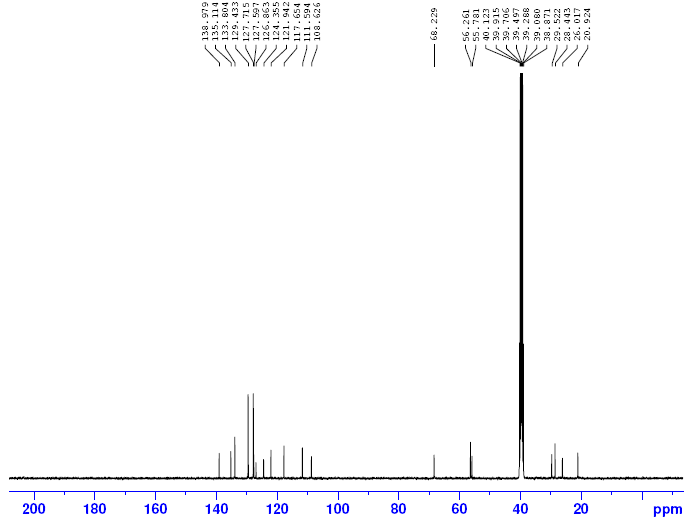


ELE-API ^13^C NMR data


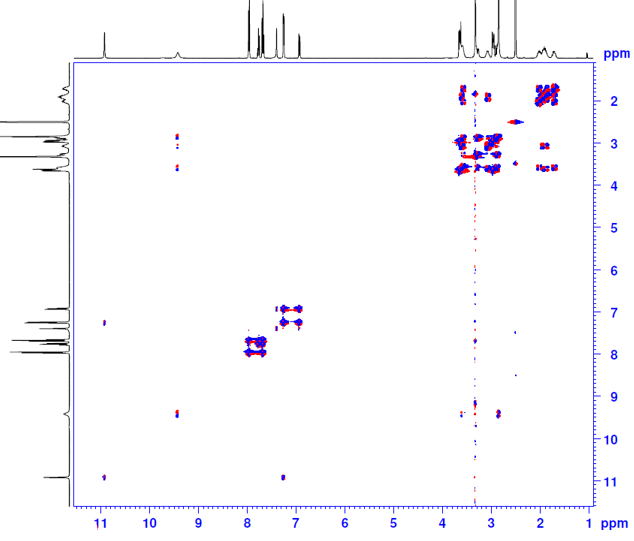


ELE-API COSY NMR data


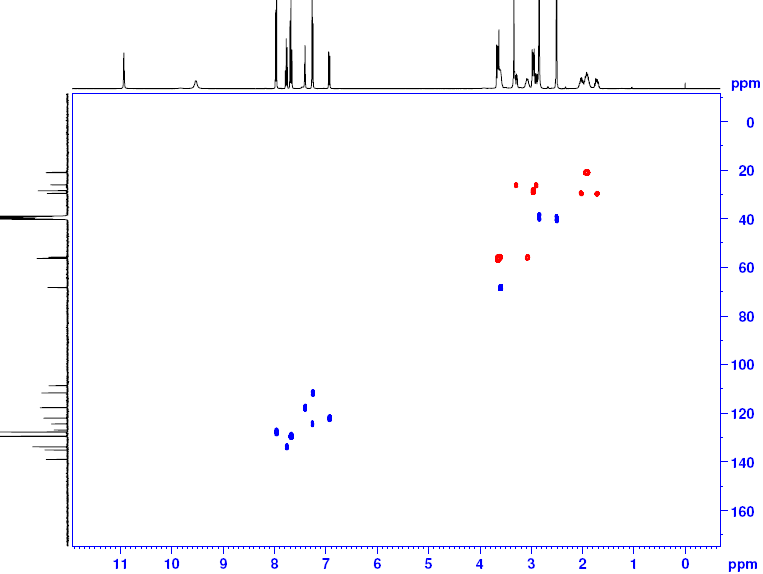


ELE-API HSQC NMR data


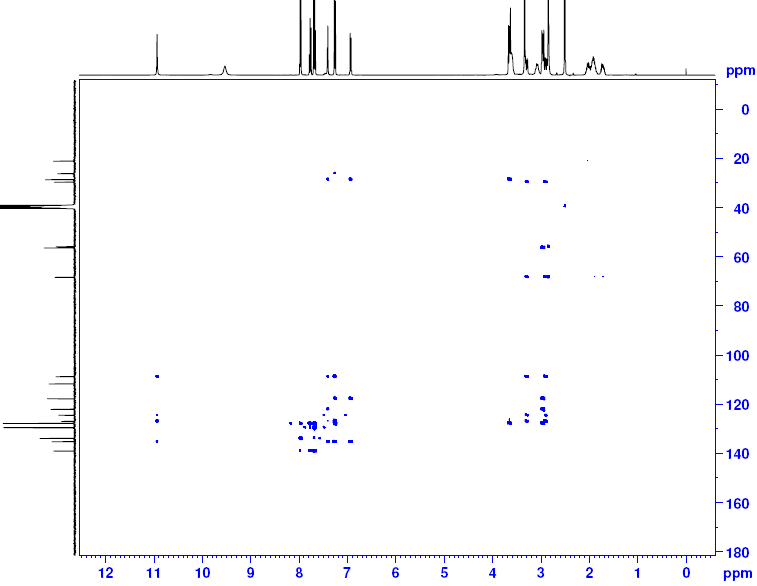


ELE-API HMBC NMR data

ELE-API HRMS data


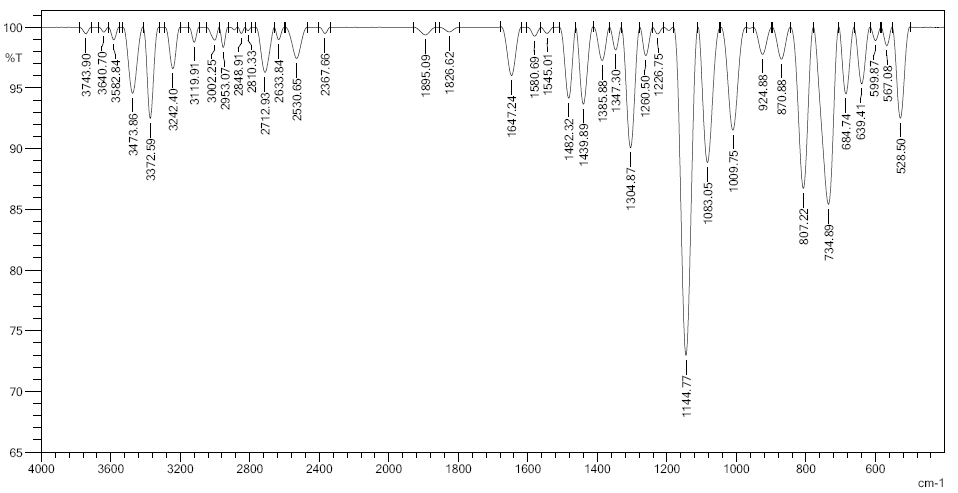


ELE-API IR data

**Figure S1: Structural characterisation data of Eletripton**

**ELE-DP-1 Analytical data:**

ELE-DP-1 Structure

Relative NMR assignments for ELE-DP-1

| **Atom No** | **Atom Symbol** | **Chemical Shift (PPM) & Coupling Const(J-Hz)** | | **Atom No** | **Atom Symbol** | **Chemical Shift (PPM) & Coupling Const(J-Hz)** | |
| --- | --- | --- | --- | --- | --- | --- | --- |
|  |  | **^1^H** | **^13^C** |  |  | **^1^H** | **^13^C** |
| 1 | C | - | 149.2 | 15 | CH_2_ | 1.38,1.61(m,2H) | 43.37 |
| 2 | C | - | 155.68 | 16 | CH | 1.48(m,1H) | 31.36 |
| 3 | CH | 7.05(s,1H) | 108.22 | 17 | CH_2_ | 1.23,1.55(m.2H) | 32.84 |
| 4 | C | - | 146.52 | 18,20 | N-CH_2_ | 2.73(m,2H), 1.90(q,12.0Hz,2H) | 53.04,53.10 |
| 5 | C | - | 126.43 | 21 | CH_2_ | 1.15,1.68(m,2H) | 33.18 |
| 6 | CH | 7.07(s,1H) | 104.51 | 22 | N-CH_2_ | 3.45(s,2H) | 62.12 |
| 7 | CH_2_ | 2.87,3.14(d,17.2Hz,2H) | 40.87 | 23 | C | - | 137.91 |
| 8 | C | - | 78.48 | 24,28 | CH | 7.28(m,2H) | 128.89 |
| 9 | CO | - | 205.33 | 25,27 | CH | 7.28(m,2H) | 128.1 |
| 11 | OCH_3_ | 3.79(s,3H) | 55.59 | 26 | CH | 7.22(m,2H) | 126.93 |
| 13 | OCH_3_ | 3.86(s,3H) | 55.93 | 29 | OH | 5.31(br peak 1H) | - |
|  |  |  |  |  |  |  |  |

**
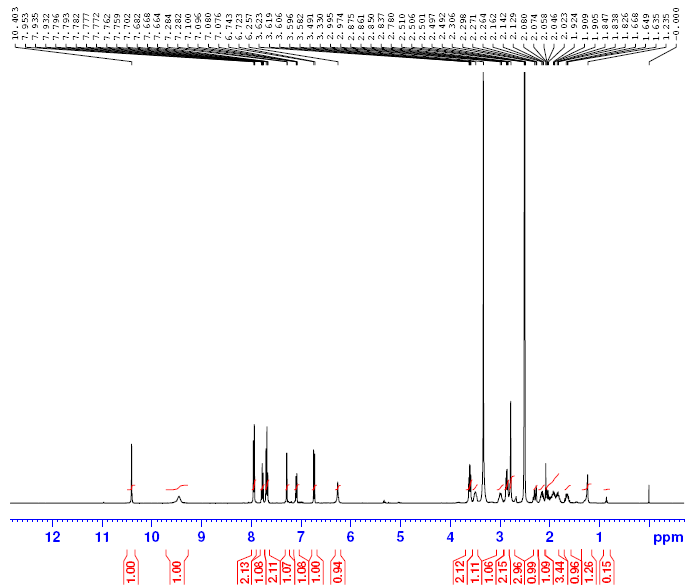
**

ELE-DP-1 ^1^H NMR data

**
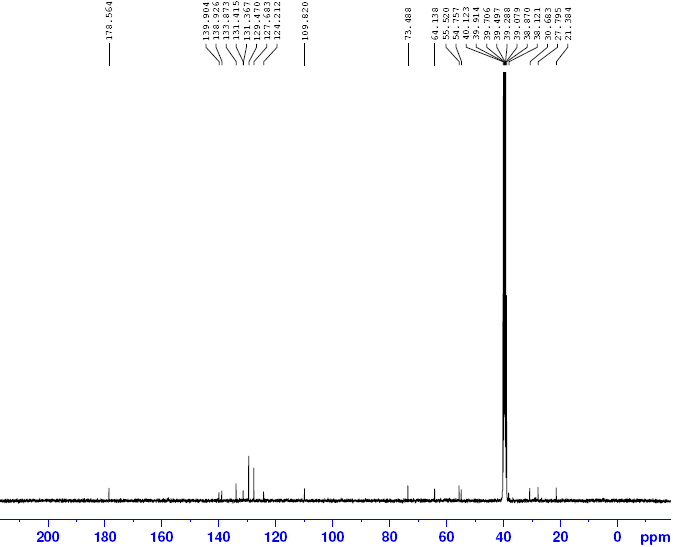
**

ELE-DP-1 ^13^C NMR data

**
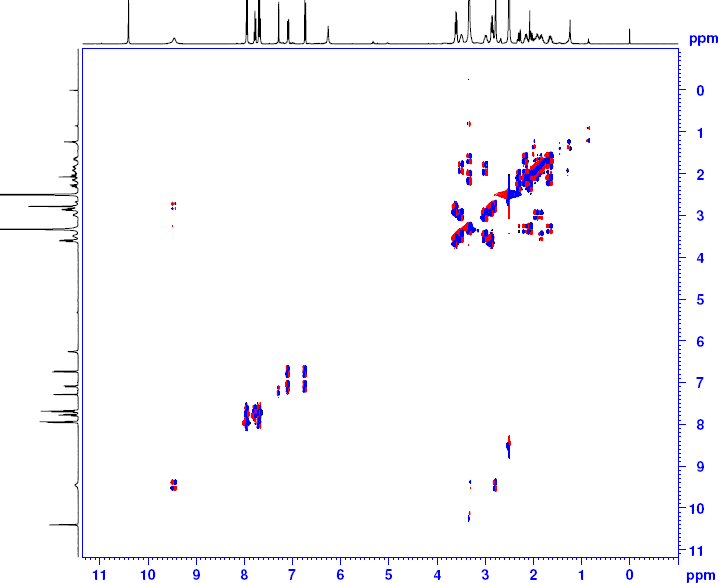
**

ELE-DP-1 COSY NMR data

**
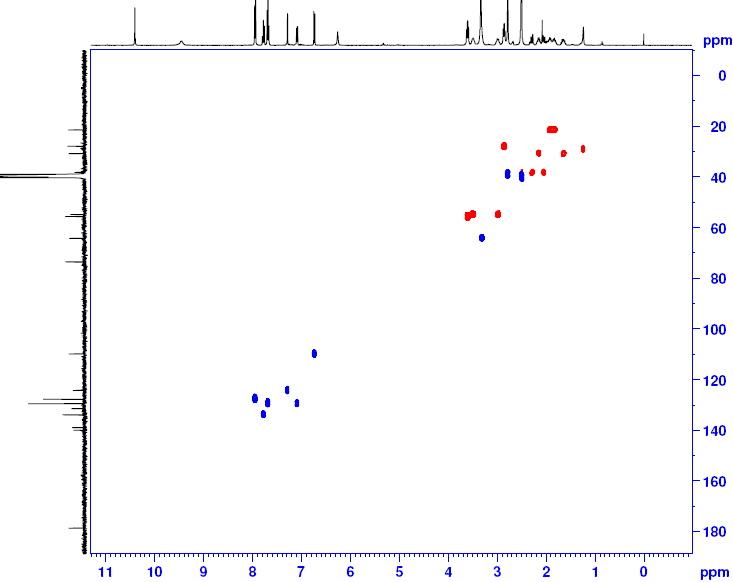
**

ELE-DP-1 HSQC NMR data


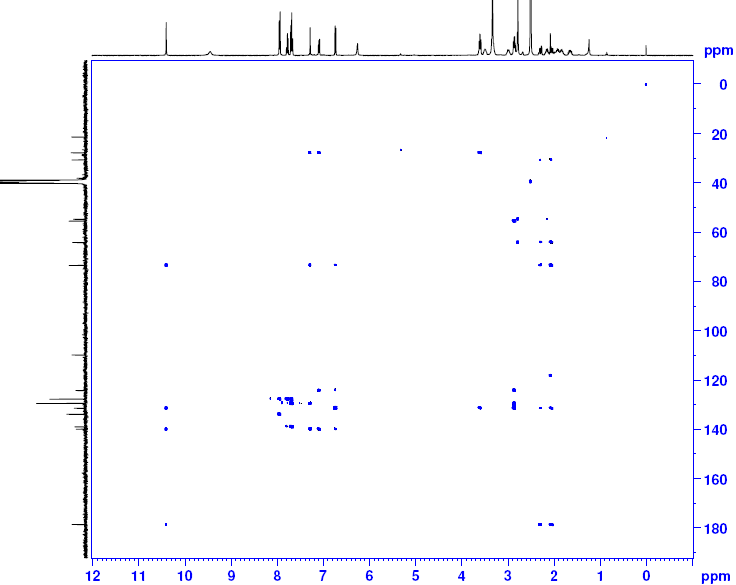


ELE-DP-1 HMBC NMR data

ELE-DP-1 ^15^N HMBC NMR data

ELE-DP-1 HRMS & HRMS-MS data

**
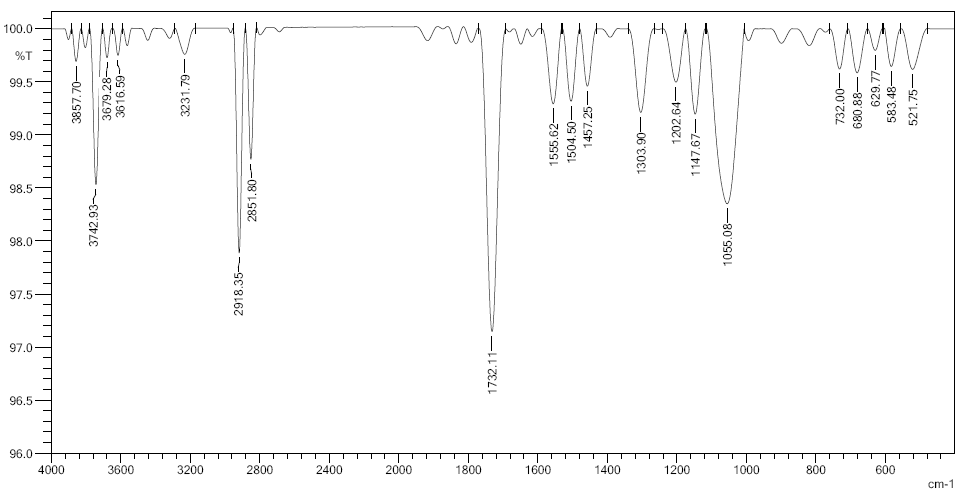
**

ELE-DP-1 IR data

**Figure S2: Structural characterisation data of ELE-DP-1**

**ELE-DP-2 Analytical data:**

ELE-DP-2 Structure

Relative NMR assignments for ELE-DP-2

| **Atom No** | **Atom Symbol** | **Chemical Shift (PPM) & Coupling Const(J-Hz)** | | **Atom No** | **Atom Symbol** | **Chemical Shift (PPM) & Coupling Const(J-Hz)** | |
| --- | --- | --- | --- | --- | --- | --- | --- |
|  |  | **^1^H** | **^13^C** |  |  | **^1^H** | **^13^C** |
| 1 | C | - | 151.29 | 13 | OCH_3_ | 3.96(s,3H) | 56.87 |
| 2 | C | - | 157.39 | 15 | CH_2_ | 2.51(d,7.2Hz,2H) | 40.68 |
| 3 | CH | 7.00(s,1H) | 107.68 | 16 | CH | 1.94(s,1H) | 34.61 |
| 4 | C | - | 135.14 | 17,21 | CH_2_ | 1.83(d,13.2Hz.2H), 1.48(q,11.6Hz,2H) | 31.5 |
| 5 | C | - | 113.86 | 18,20 | N-CH_2_ | 3.15(d,11.6Hz,2H), 2.48(11.2Hz,2H) | 54.13 |
| 6 | CH | 7.55(s.1H) | 110.1 | 22 | N-CH_2_ | 3.86(s,2H) | 63.24 |
| 7 | CH | 6.46(s,1H) | 105.95 | 23 | C | - | 135.14 |
| 8 | C | - | 155.99 | 24,28 | CH | 7.40(m,2H) | 131.63 |
| 9 | CO | - | 164.95 | 25,27 | CH | 7.40(m,2H) | 129.89 |
| 11 | OCH_3_ | 3.92(s,3H) | 56.69 | 26 | CH | 7.37(m,1H) | 129.77 |
|  |  |  |  |  |  |  |  |


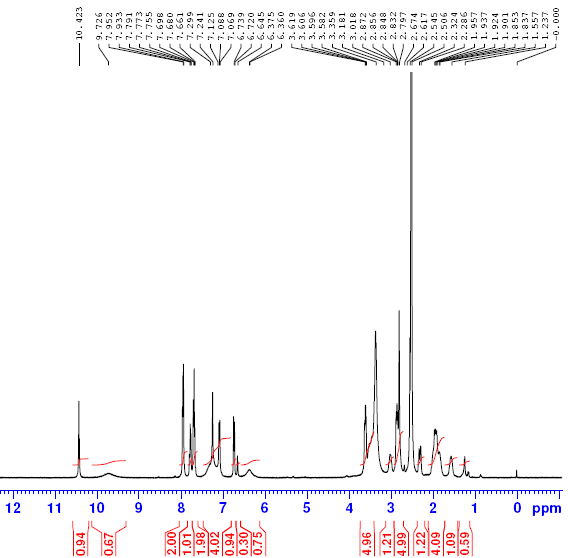


ELE-DP-2 ^1^H NMR data

**
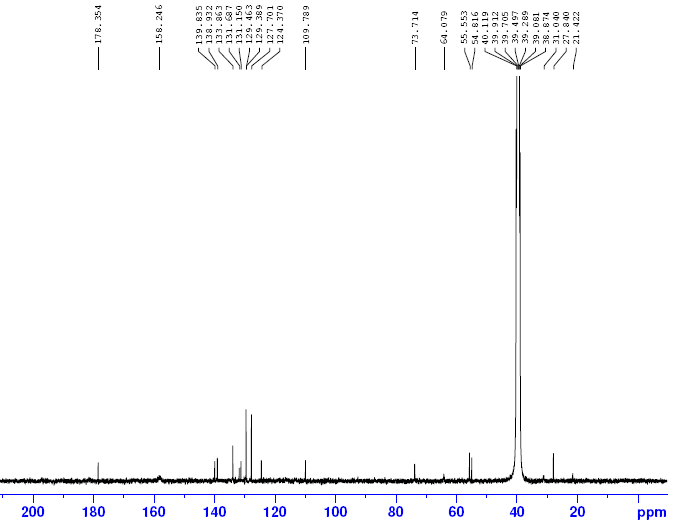
**

ELE-DP-2 ^13^C NMR data

**
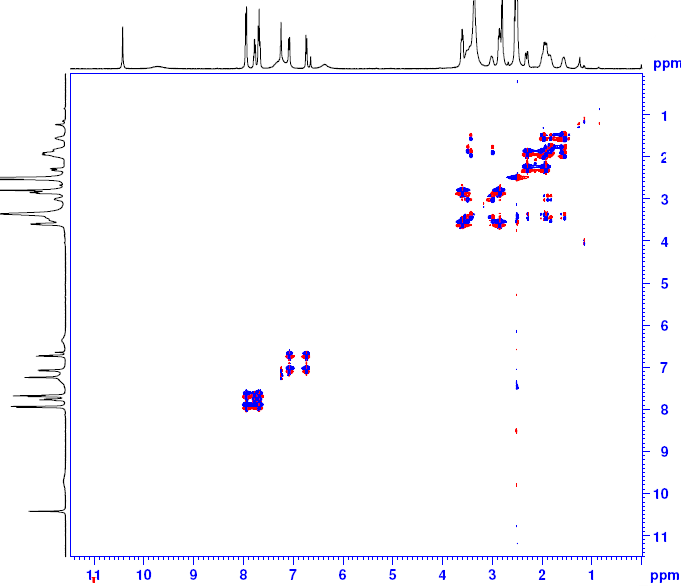
**

ELE-DP-2 COSY NMR data

**
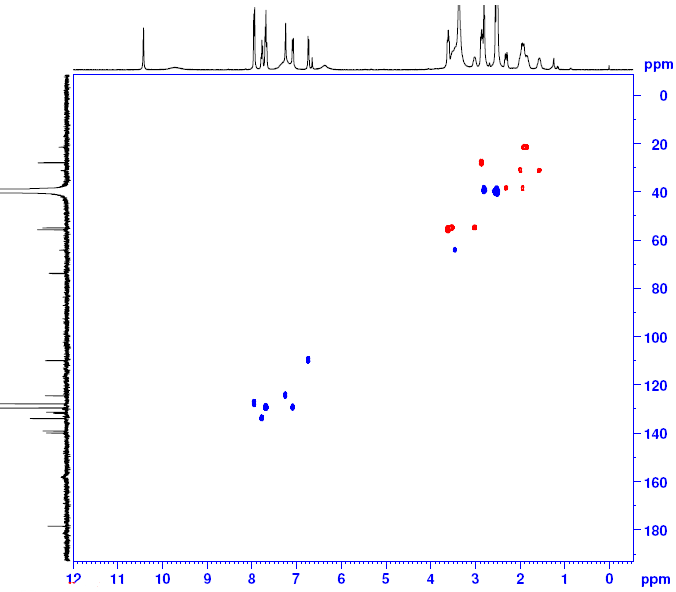
**

ELE-DP-2 HSQC NMR data

**
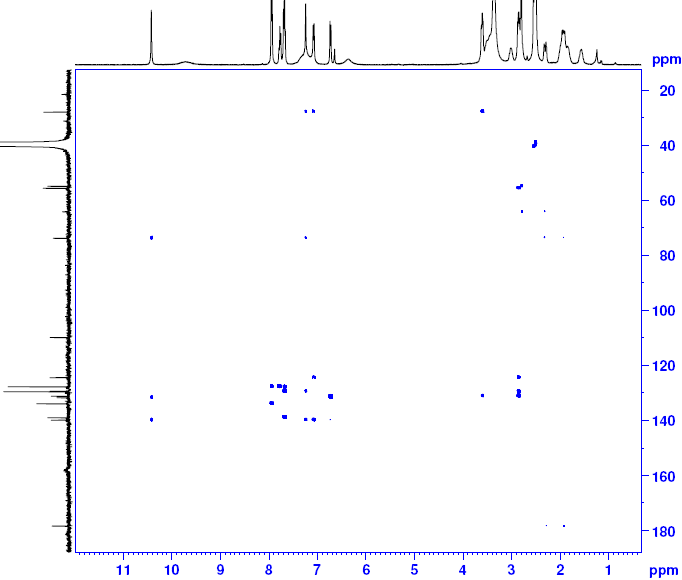
**

ELE-DP-2 HMBC NMR data

ELE-DP-2 HRMS data


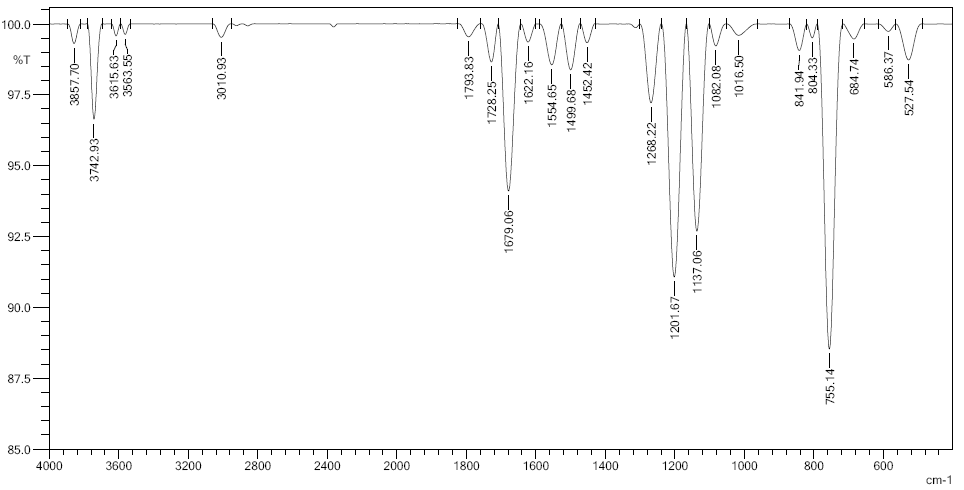


ELE-DP-2 IR data

**Figure S3: Structural characterisation data of ELE-DP-2**

**ELE-DP-3 Analytical data:**

ELE-DP-3 Structure

Relative NMR assignments for ELE-DP-3

| **Atom No** | **Atom Symbol** | **Chemical Shift (PPM) & Coupling Const(J-Hz)** | | **Atom No** | **Atom Symbol** | **Chemical Shift (PPM) & Coupling Const(J-Hz)** | |
| --- | --- | --- | --- | --- | --- | --- | --- |
|  |  | **^1^H** | **^13^C** |  |  | **^1^H** | **^13^C** |
| 1 | C | - | 146.73 | 15 | CH_2_ | 2.39(d,4.80Hz,2H) | 48.17 |
| 2 | C | - | 151.41 | 16 | CH | 1.75(m,1H) | 30.97 |
| 3 | CH | 6.84(s,1H) | 115.53 | 17,21 | CH_2_ | 1.60(d,12Hz,2H), 1.18(q,11.6Hz,2H) | 31.49 |
| 4 | C | - | 131.39 | 18,20 | N-CH_2_ | 1.98,2.76(br peaks,4H) | 52.92 |
| 5 | C | - | 121.49 | 22 | N-CH_2_ | 3.47(s,2H) | 62.13 |
| 6 | CH | 7.43(s.1H) | 113.47 | 23 | C | - | 137.87 |
| 7 | CH_2_ | 4.00(s,2H) | 48.17 | 24,28 | CH | 7.32(m,2H) | 128.9 |
| 8 | CO | - | 206.61 | 25,27 | CH | 7.32(m,2H) | 128.13 |
| 9 | CO | - | 167.64 | 26 | CH | 7.26(m,1H) | 126.97 |
| 11 | OCH_3_ | 3.77(s,3H) | 55.48 | 29 | OH | 12.45(broad hump) | - |
| 13 | OCH_3_ | 3.79(s,3H) | 55.61 | 13 | OCH_3_ | 3.79(s,3H) | 55.61 |
|  |  |  |  |  |  |  |  |

**
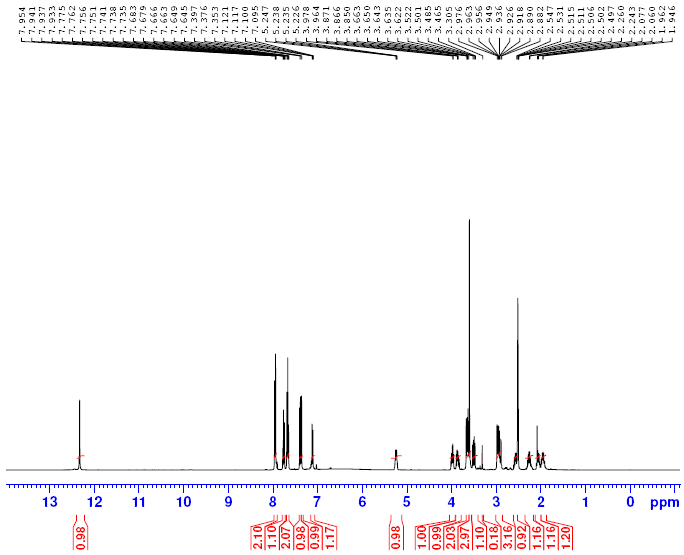
**

ELE-DP-3 ^1^H NMR data

**
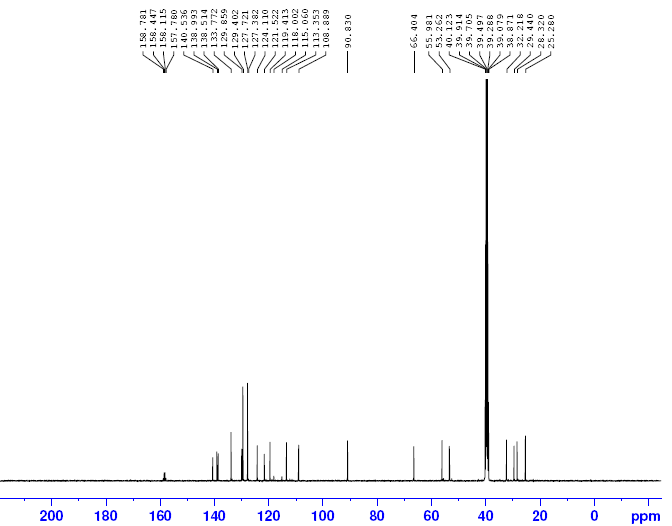
**

ELE-DP-3 ^13^C NMR data

**
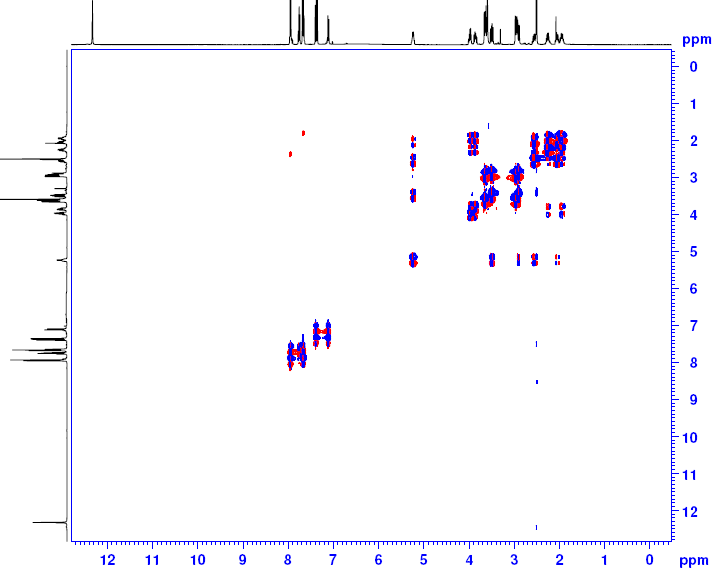
**

ELE-DP-3 COSY NMR data

**
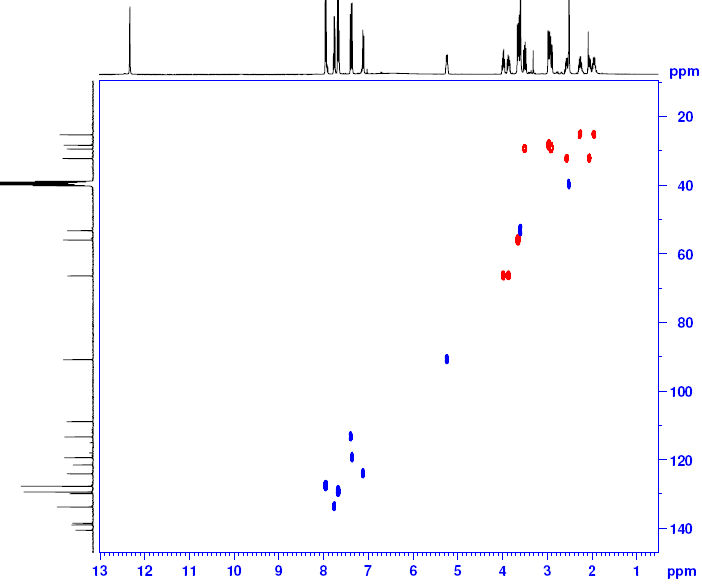
**

ELE-DP-3 HSQC NMR data

**
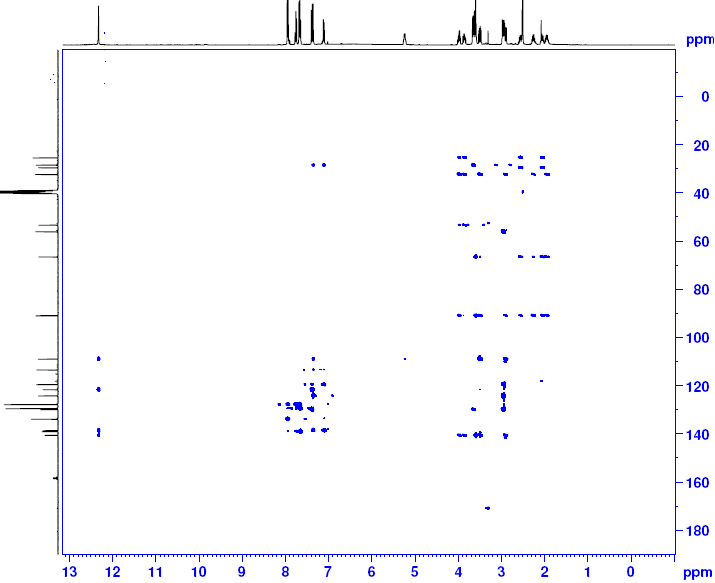
**

ELE-DP-3 HMBC NMR data

ELE-DP-3 HRMS data

**
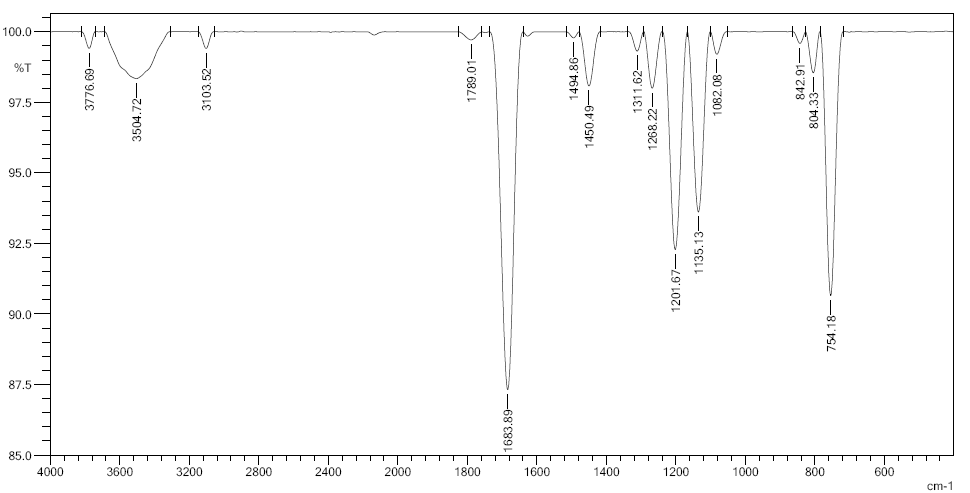
**

ELE-DP-3 IR data

**Figure S4: Structural characterisation data of ELE-DP-3**

**ELE-DP-4 Analytical data:**

ELE-DP-4 Structure

Relative NMR assignments for ELE-DP-4

| **Atom No** | **Atom Symbol** | **Chemical Shift (PPM) & Coupling Const(J-Hz)** | | **Atom No** | **Atom Symbol** | **Chemical Shift (PPM) & Coupling Const(J-Hz)** | |
| --- | --- | --- | --- | --- | --- | --- | --- |
|  |  | **^1^H** | **^13^C** |  |  | **^1^H** | **^13^C** |
| 1 | C | - | 146.73 | 15 | CH_2_ | 2.39(d,4.80Hz,2H) | 48.17 |
| 2 | C | - | 151.41 | 16 | CH | 1.75(m,1H) | 30.97 |
| 3 | CH | 6.84(s,1H) | 115.53 | 17,21 | CH_2_ | 1.60(d,12Hz,2H), 1.18(q,11.6Hz,2H) | 31.49 |
| 4 | C | - | 131.39 | 18,20 | N-CH_2_ | 1.98,2.76(br peaks,4H) | 52.92 |
| 5 | C | - | 121.49 | 22 | N-CH_2_ | 3.47(s,2H) | 62.13 |
| 6 | CH | 7.43(s.1H) | 113.47 | 23 | C | - | 137.87 |
| 7 | CH_2_ | 4.00(s,2H) | 48.17 | 24,28 | CH | 7.32(m,2H) | 128.9 |
| 8 | CO | - | 206.61 | 25,27 | CH | 7.32(m,2H) | 128.13 |
| 9 | CO | - | 167.64 | 26 | CH | 7.26(m,1H) | 126.97 |
| 11 | OCH_3_ | 3.77(s,3H) | 55.48 | 29 | OH | 12.45(broad hump) | - |
| 13 | OCH_3_ | 3.79(s,3H) | 55.61 | 13 | OCH_3_ | 3.79(s,3H) | 55.61 |
|  |  |  |  |  |  |  |  |

**
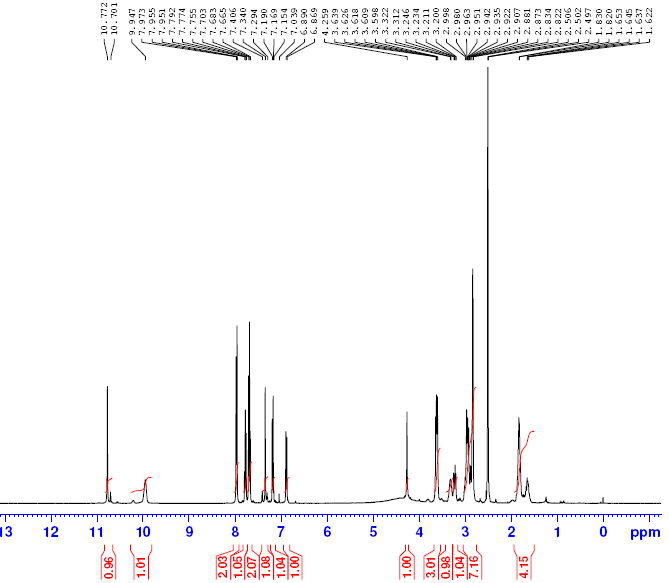
**

ELE-DP-4 ^1^H NMR data

**
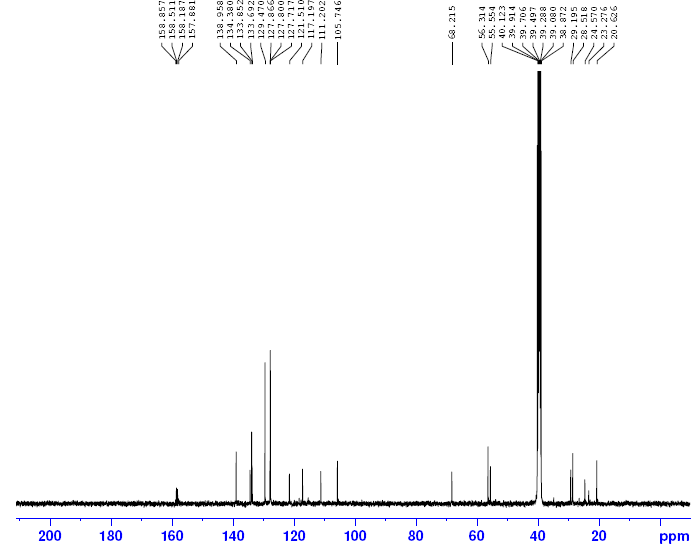
**

ELE-DP-4 ^13^C NMR data-1

**
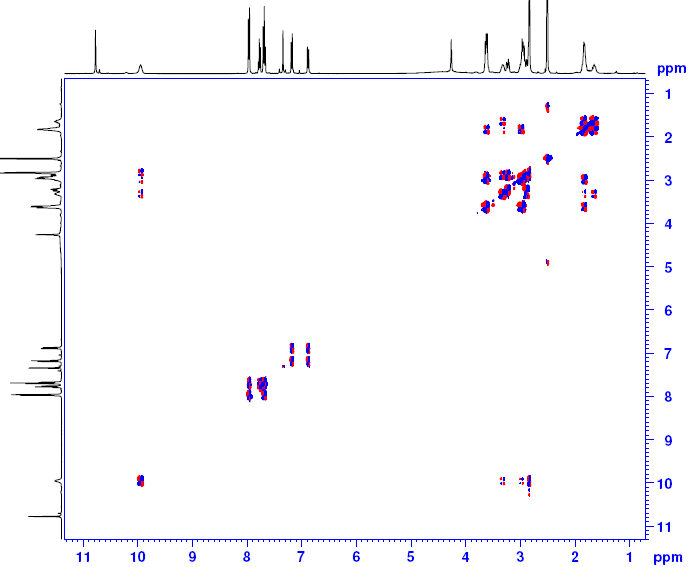
**

ELE-DP-4 COSY NMR data

**
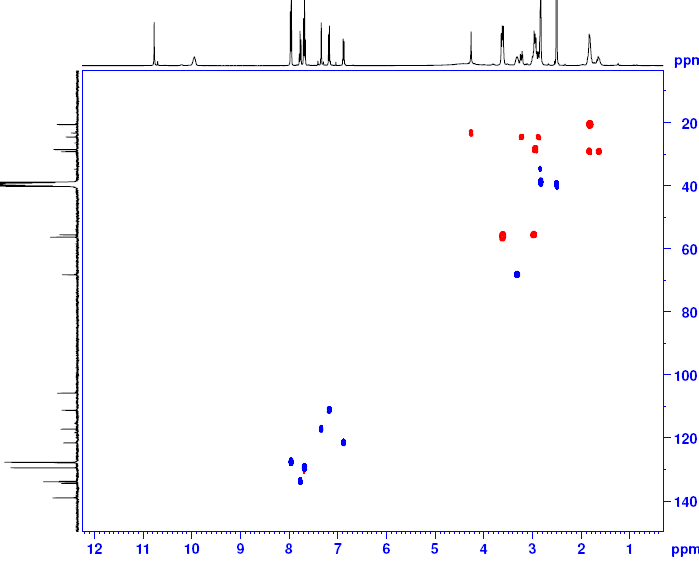
**

ELE-DP-4 HSQC NMR data

**
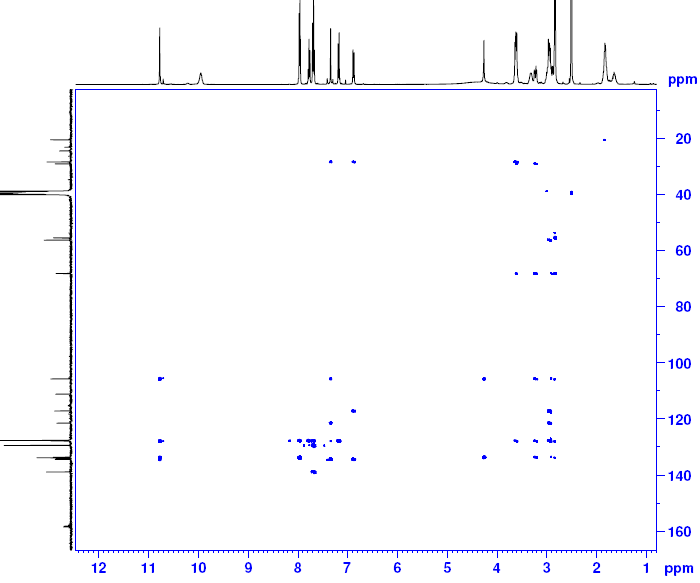
**

ELE-DP-4 HMBC NMR data

ELE-DP-3 HRMS data


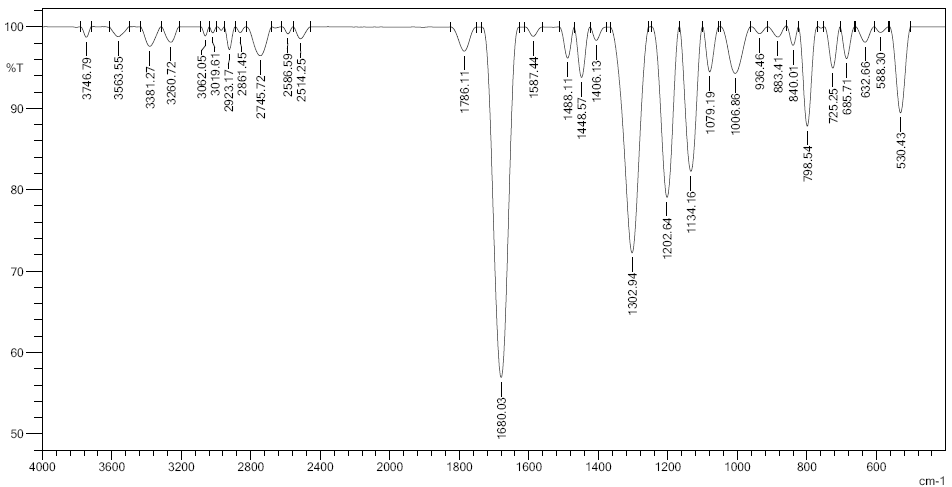


ELE-DP-3 IR data

**Figure S5: Structural characterisation data of ELE-DP-4**
